# Drug interactions between hormonal contraceptives and antiretrovirals

**DOI:** 10.1097/QAD.0000000000001392

**Published:** 2017-04-03

**Authors:** Kavita Nanda, Gretchen S. Stuart, Jennifer Robinson, Andrew L. Gray, Naomi K. Tepper, Mary E. Gaffield

**Affiliations:** a FHI 360, Durham; b University of North Carolina Medical School, Chapel Hill; c Johns Hopkins University School of Medicine, Baltimore, Maryland, USA; d Division of Pharmacology, Discipline of Pharmaceutical Sciences, University of KwaZulu-Natal, Durban, South Africa; e Division of Reproductive Health, U.S. Centers for Disease Control and Prevention, Atlanta, Georgia, USA; f Department of Reproductive Health and Research, World Health Organization, Geneva, Switzerland.

**Keywords:** antiretroviral therapy, contraceptive implant, depot medroxyprogesterone acetate, HIV, hormonal contraception, systematic review

## Abstract

**Objective:**

To summarize published evidence on drug interactions between hormonal contraceptives and antiretrovirals.

**Design:**

Systematic review of the published literature.

**Methods:**

We searched PubMed, POPLINE, and EMBASE for peer-reviewed publications of studies (in any language) from inception to 21 September 2015. We included studies of women using hormonal contraceptives and antiretrovirals concurrently. Outcomes of interest were effectiveness of either therapy, toxicity, or pharmacokinetics. We used standard abstraction forms to summarize and assess strengths and weaknesses.

**Results:**

Fifty reports from 46 studies were included. Most antiretrovirals whether used for therapy or prevention, have limited interactions with hormonal contraceptive methods, with the exception of efavirenz. Although depot medroxyprogesterone acetate is not affected, limited data on implants and combined oral contraceptive pills suggest that efavirenz-containing combination antiretroviral therapy may compromise contraceptive effectiveness of these methods. However, implants remain very effective despite such drug interactions. Antiretroviral plasma concentrations and effectiveness are generally not affected by hormonal contraceptives.

**Conclusion:**

Women taking antiretrovirals, for treatment or prevention, should not be denied access to the full range of hormonal contraceptive options, but should be counseled on the expected rates of unplanned pregnancy associated with all contraceptive methods, in order to make their own informed choices.

## Introduction

Women living with HIV will likely take combination antiretroviral therapy (cART) for much of their lives [1]. Those at high risk for HIV may also use antiretrovirals for preexposure prophylaxis (PrEP). Contraceptive use among women living with HIV or using antiretrovirals for PrEP is critical, as unintended pregnancy and short interpregnancy intervals can be associated with negative health consequences for both mother and infant [2–4]. Decreasing unintended pregnancies also reduces vertical HIV transmission [5]. Hormonal contraceptives are highly used worldwide, including in areas of high HIV prevalence; they are also among the most effective contraceptive methods [6,7]. Evidence-based guidance for hormonal contraceptives use among women using cART or PrEP is needed to ensure access to a full range of the best contraceptive methods, and therefore increase the likelihood of achieving their reproductive life planning goals.

Concurrent use of hormonal contraceptives and antiretrovirals can lead to drug interactions, predominantly due to effects on liver metabolism (Tables 1 and 2). In the liver, cytochrome P450 (CYP) enzymes catalyze many important reactions, with the most significant for contraceptive metabolism being CYP3A4, which is also expressed in the intestines [8,9]. Antiretrovirals include different classes of drug (Table 2), including nonnucleoside reverse transcriptase inhibitors (NNRTIs), nucleoside analogue reverse transcriptase inhibitors or nucleotide analogue reverse transcriptase inhibitor (NRTIs), protease inhibitors, fusion inhibitors, and integrase inhibitors. The NNRTIs and integrase inhibitors are generally not substrates, inhibitors, nor inducers of cytochrome P450 enzymes [10]. In contrast, both protease inhibitors and NNRTIs are metabolized by CYP3A4 and also inhibit or induce this enzyme, resulting in increases or decreases in the concentration of concomitantly administered drugs [10].

Such interactions could lead to decreased contraceptive effectiveness (increasing risk of unintended pregnancy), decreased cART effectiveness (associated with resistance and/or HIV disease progression), decreased efficacy of PrEP (increasing risk of HIV acquisition), or increased antiretroviral or contraceptive toxicity. Based on theoretical concerns and limited data, women using cART are sometimes offered fewer contraceptive choices than their HIV-negative peers [11]. The objective of this review was to systematically examine published evidence on drug interactions between hormonal contraceptives and antiretrovirals, in order to contribute to improved clinical and policy decision-making.

## Methods

We followed the PRISMA and MOOSE guidelines for conducting the review and reporting the results [12,13].

We searched PubMed, POPLINE, and EMBASE from database inception to 21 September 2015 for studies of hormonal contraceptive and antiretroviral drug interactions (Supplement 1). We also hand-searched reference lists of published studies, and contacted topic experts.

### Study selection

We included published studies of women using hormonal contraceptives (Table 1), including combined oral contraceptives (COCs), progestin-only pills (POPs), emergency contraceptive pills (ECPs), injectables, vaginal rings, patches, or implants. Studies included women who were either HIV-positive, HIV-negative but at risk of HIV, or healthy, who concurrently used cART, PrEP, or single antiretrovirals and hormonal contraceptives. We included studies reporting on women taking oral contraceptives where the type of oral contraceptive was not specified. We excluded studies evaluating women on cART without comparisons by contraceptive use, those evaluating only genital HIV viral load, and those evaluating only hormonal intrauterine devices (IUDs). We also excluded case or case-series reports, cross-sectional studies, reviews, editorials, and letters.

Outcomes of interest were clinical and pharmacokinetic measures of the contraceptive and the antiretroviral. Clinical outcomes included measures of contraceptive, cART, or PrEP effectiveness, and combined toxicity. Contraceptive effectiveness measures of interest were pregnancy or surrogate measures of pregnancy risk, including ovulation, ovarian activity, or cervical mucus. Because no studies reported on true ovulation as documented by ultrasound, we included studies using serum progesterone alone as a marker of presumed ovulation. For cART effectiveness, we included studies that reported markers of HIV disease progression such as CD4^+^ cell count or HIV viral load, need for change in cART regimen, or death; for PrEP effectiveness, the relevant outcome was HIV prevention. Pharmacokinetic endpoints were plasma drug concentrations over time, as well as the area under the concentration–time curve (AUC), half-life (*t*_1/2_), minimum (*C*_min_; trough) and maximum (*C*_max_; peak) concentrations, for both contraceptive steroids and antiretrovirals.

### Data abstraction and management

After screening and removal of duplicates, we abstracted relevant data from each included report using a predesigned form. Two authors independently reviewed selected manuscripts, with differences resolved by consensus.

We described strengths, weaknesses, and funding source for each included study (Tables 3 and 4) [14–65], but did not do formal quality assessment because no formal evidence grading system exists for pharmacokinetic studies.

## Results

**Fig. 1:**
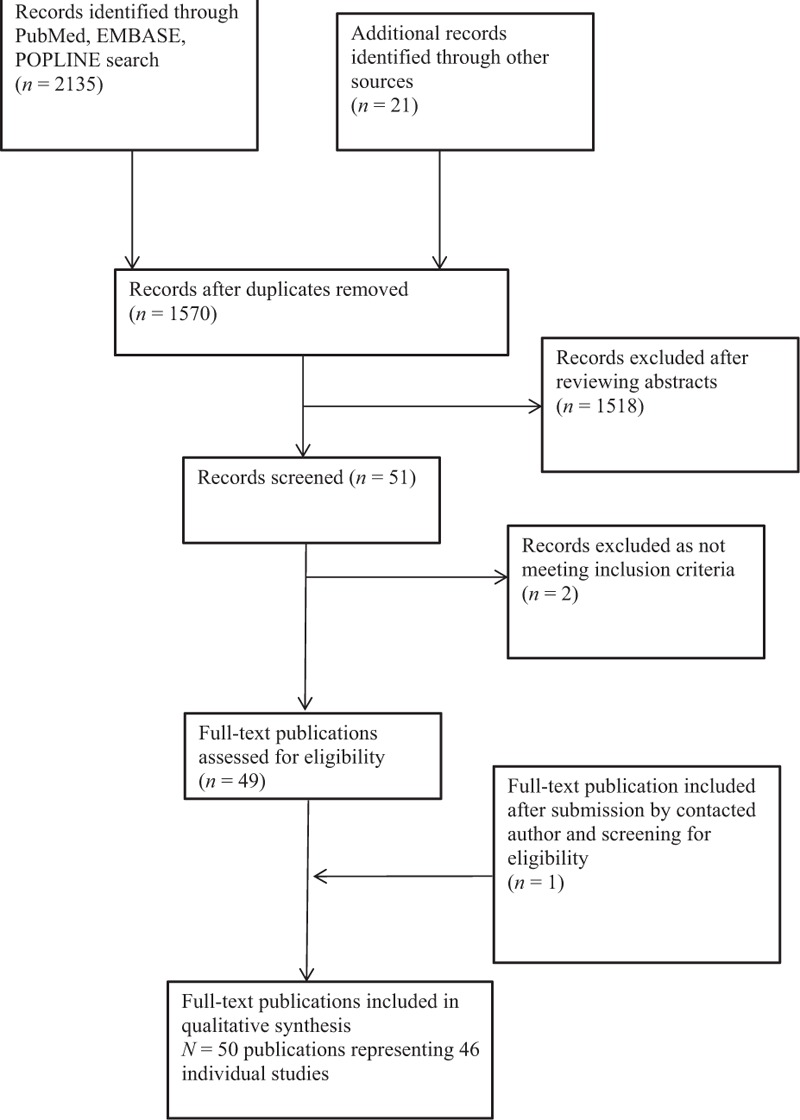
Flow diagram of publication selection for inclusion into the review.

Our search identified 1570 records. Fifty published reports from 46 individual studies met the inclusion criteria (Fig. 1, Tables 3 and 4). Four reports were secondary analyses or subsets of the primary studies and are included with the primary study in the tables [14–17]. The results are presented by outcome assessed, focusing first on the most important clinical outcomes (contraceptive effectiveness, antiretroviral effectiveness, toxicity associated with combined administration), then the pharmacokinetic data (for contraceptives and antiretrovirals), in each case by antiretroviral class and by contraceptive method.

### Contraceptive effectiveness

Although pregnancy is the most relevant outcome, few large studies were designed to investigate contraceptive effectiveness. Several secondary analyses helped fill this gap, particularly for women using nevirapine-containing or efavirenz-containing cART. Although some small pharmacokinetic studies of healthy women report on pregnancy, women were generally required to use additional contraception; these studies are included in Table 3 but not summarized here.

### Nonnucleoside reverse transcriptase inhibitors

Fourteen reports from clinical trials and six secondary analyses described contraceptive effectiveness measures among women using NNRTIs and hormonal contraceptives (Table 3).

#### Oral contraceptives

Two clinical trials of women using cART and oral contraceptives [18,19], six secondary analyses [20–25] and five pharmacokinetic trials (mostly in healthy women using single antiretrovirals with COCs) [26–30], evaluated pregnancy or ovulation. No pregnancies were found to be associated with nevirapine or efavirenz in the prospective clinical trials.

Pregnancy rates and ovulation rates did not differ between HIV-positive women taking COCs and nevirapine-containing cART and those not yet taking cART [18]. In a small trial of women using COCs with efavirenz-containing cART, three women ovulated (out of 25) but no pregnancies were reported [19]. Five small pharmacokinetic trials of NNRTIs and COCs also demonstrated no ovulation among study participants [26–30].

In large cohort studies, pregnancy rates were slightly higher among women taking efavirenz-containing cART (11–15/100 woman-years) compared with women taking oral contraceptive and nevirapine-containing cART or no cART (pregnancy rates 6–11/100 woman-years) [24,25]. Notably the reported pregnancy rates in the large cohort studies are still lower than an expected pregnancy rate of 40 per 100 woman-years among women not using any modern contraceptive and trying to prevent pregnancy.

Other retrospective cohort studies reported pregnancy rates among oral contraceptive users ranging from 2.6 to 5.8 per 100 woman-years (most, but not all, women were using nevirapine) [20,21].

#### Depot medroxyprogesterone acetate

In two pharmacokinetic studies, women using depot medroxyprogesterone acetate (DMPA) remained anovulatory when using cART containing either efavirenz or nevirapine [14,31,32]. Five cohort studies also presented pregnancy rates with injectables among cART users. In the largest, pregnancy rates ranged from 8 to 10 per 100 woman-years for injectable users, with higher rates in efavirenz users and those not on cART compared with pregnancy rates in those using nevirapine [24]. Another found women using DMPA had pregnancy rates from 3 to 5 per 100 woman-years, with lower rates among users of nevirapine and efavirenz compared with no cART [25]. Two additional studies reported pregnancy rates from 1.8 to 4.2 per 100 woman-years among DMPA users taking various antiretrovirals (primarily nevirapine) [20,21].

#### Implants

Pregnancy rates among users of cART and contraceptive implants differed by whether the implant contained levonorgestrel or etonogestrel, and whether women were taking efavirenz or nevirapine. Pregnancy rates were higher among women using the levonorgestrel implant concomitantly with efavirenz. Two prospective studies (*N* = 79 and *N* = 45) reported no pregnancies through 3 years and 6 months, respectively, among women using etonogestrel implants and NNRTI-containing cART [33,34], although the second study found that women taking efavirenz-containing cART had a 2.8% presumed ovulation rate over 6 months. In a small pharmacokinetic study of women using levonorgestrel implants and cART containing efavirenz or nevirapine, three pregnancies (3/20; 15%) occurred within 48 weeks, all in women taking efavirenz [35]. Similar findings were seen in a large retrospective study, where 15 of 121 (12.4%) women using levonorgestrel implants and efavirenz-containing cART became pregnant, at a mean duration of 16.4 months; no pregnancies occurred among women taking nevirapine-containing cART [36].

Two secondary analyses described pregnancy rates with implant use in women using efavirenz-containing or nevirapine-containing cART [24,25]. In the first, pregnancy rates for users of efavirenz, nevirapine, and no cART were 5.5, 2.3, and 3.4 per 100 woman-years for etonogestrel implant users, and 7.1, 1.9, and 3.3 per 100 woman-years for levonorgestrel implant users, respectively [24]. In the second, pregnancy rates per 100 woman-years were 1.4 for unspecified implant users not taking cART, 0 for nevirapine users, and 6 for women taking efavirenz [25].

### Protease inhibitors

Seven small pharmacokinetic trials described contraceptive effectiveness measures among women using protease inhibitors and hormonal contraceptives (Table 3) [14,16,31,34,37–39].

#### Combined oral contraceptives/patches

Co-administration of darunavir/ritonavir with COCs resulted in no ovulation [38]. Similarly, coadministration of lopinavir/ritonavir containing cART in women using a contraceptive patch also found no ovulations [37].

#### Progestin-only pills

One report showed that oral norethindrone thickened cervical mucus and led to similar mucus scores in women using protease inhibitor-containing cART compared with those taking NRTIs alone [16].

#### Depot medroxyprogesterone acetate

Reports of women using lopinavir/ritonavir-containing or nelfinavir-containing cART with DMPA found that no women ovulated [14,31,39].

#### Implants

No ovulations nor pregnancies were reported in a pharmacokinetic study of etonogestrel implant users taking lopinavir/ritonavir-containing cART [34].

### Nucleoside/nucleotide reverse transcriptase inhibitors

Analyses of two large trials of hormonal contraceptives and NRTIs used for PrEP found that use of tenofovir/emtricitabine did not affect pregnancy rates among users of COCs, injectables, or implants [40,41].

#### Integrase inhibitors

A small pharmacokinetic study of dolutegravir with COCs resulted in no ovulations [42].

### Antiretroviral effectiveness

Eight reports evaluated the effects of hormonal contraceptive use on the effectiveness of NNRTI-containing or protease inhibitor-containing cART, and found no effects on death, CD4^+^ cell count, or plasma viral load with concurrent use of DMPA, levonorgestrel implants, or oral contraceptives [14,43–49]. Use of DMPA also did not affect the efficacy of PrEP [50].

### Toxicity of combined administration

Studies among healthy women using hormonal contraceptives concurrently with single antiretrovirals, or HIV-positive women using cART, generally reported no difference in adverse events of concurrent treatment compared with use of either hormonal contraceptives or antiretrovirals alone (Table 3). One HIV prevention trial evaluated pharmacodynamic interactions between tenofovir-containing PrEP with oral contraceptives or DMPA, and found that bone mineral density was not significantly decreased [51].

### Contraceptive pharmacokinetics

Thirty-two reports include contraceptive pharmacokinetic measures among women using antiretrovirals and hormonal contraceptives (Table 4).

### Nonnucleoside reverse transcriptase inhibitors

Contraceptive pharmacokinetics among women using NNRTIs and hormonal contraceptives were described in 11 studies (Table 4) [15,26–32,34,52,53].

#### Combined oral contraceptives

Two studies evaluated efavirenz with COCs: one in women taking only efavirenz, and one in women taking efavirenz-containing cART [15,26]. Ethinyl estradiol concentrations were not significantly changed, but progestin levels decreased by approximately 60%.

Three studies reported the effect of nevirapine on COC pharmacokinetics. Ethinyl estradiol levels varied from being unchanged to being approximately 30–60% lower [15,30,53]. Progestin levels were not significantly affected.

Studies of etravirine and rilpivirine in women taking COCs found minimal effects [27,28]. Similarly, a study of fosdevirine (the development of which was discontinued due to toxicity) found no effect on hormone levels in COC users [29].

#### Depot medroxyprogesterone acetate

Two studies evaluated the effect of NNRTIs on DMPA, and found no difference in medroxyprogesterone acetate pharmacokinetics through 12 weeks with concurrent use of either efavirenz-containing or nevirapine-containing cART [31,32].

#### Implants

A study of women using etonogestrel implants found 54–70% lower etonogestrel levels among women taking efavirenz-containing cART compared with women taking no cART [34].

#### Emergency contraceptive pills

One study in healthy women showed that levonorgestrel levels were 56% lower in ECP users after use of efavirenz [52].

### Protease Inhibitors

Ten reports described contraceptive pharmacokinetics among women using protease inhibitors and hormonal contraceptives (Table 3) [17,31,34,37–39,54–57].

#### Combined oral contraceptives/Patch

Two studies examining concurrent use of ritonavir and COCs found decreased ethinyl estradiol levels, whereas progestin levels were unaffected [54,57]. Another study showed decreased ethinyl estradiol levels, but increased progestin levels, when COCs were used with atazanavir/ritonavir [55]. Similar findings were reported with lopinavir/ritonavir-containing cART and the contraceptive patch [37]. This study also reported lower ethinyl estradiol levels with concurrent use of a single COC pill, but progestin levels were not evaluated. Only darunavir/ritonavir was associated with significantly lower ethinyl estradiol levels as well as slightly lower norethindrone levels [38].

#### Progestin-only pills

In women receiving protease inhibitor-containing cART, norethindrone levels were higher compared with controls [56]. A subanalysis restricted to women using ritonavir-boosted atazanavir confirmed this finding [17].

#### Depot medroxyprogesterone acetate

One study showed significantly increased medroxyprogesterone acetate concentrations, compared with historical controls, in women using DMPA and lopinavir/ritonavir-containing cART [39].

#### Implants

A study evaluating the effect of cART on the pharmacokinetics of the etonogestrel implant found women using lopinavir/ritonavir-containing cART had etonogestrel levels approximately 50% higher than women not taking cART [34].

### Nucleoside/nucleotide reverse transcriptase inhibitors

Two studies evaluating NRTIs used for PrEP with COCs or levonorgestrel implants showed no change in hormone levels [58,59].

### Chemokine receptor 5 antagonists

Two studies showed that neither maraviroc nor vicriviroc impacted hormone levels when used concurrently with COCs [57,60].

### Integrase inhibitors

In two studies, concurrent use of COCs and raltegravir led to small increases in progestin exposure [61], but dolutegravir had no impact on hormone levels [42].

### Antiretroviral pharmacokinetics

Fifteen studies described antiretroviral pharmacokinetics among women using antiretrovirals and hormonal contraceptives; most were among healthy women and compared drug concentrations to historical controls (Table 4) [19,26,27,31,37–39,53–55,60,62–65].

### Nonnucleoside reverse transcriptase inhibitors

Three studies evaluated the impact of COCs on the pharmacokinetics of efavirenz [19,26,62]. Among women using COCs and efavirenz alone, concentrations were similar to historical controls [26]. However, in a trial of women on efavirenz-containing cART, use of COCs led to efavirenz concentrations lower than historical controls [19]. Another analysis of women taking efavirenz-containing cART found no difference in efavirenz concentrations between hormonal contraceptive users and nonusers [62].

Three studies evaluated the impact of COCs on nevirapine levels. In two reports of women using nevirapine-containing cART, nevirapine levels were not significantly different in women using COCs [19,53]. Time to undetectable nevirapine levels was longer in women receiving single-dose nevirapine and using COCs [63]. Another study found rilpivirine levels in COC users to be similar to historical controls [27].

Among women on various cART regimens, nevirapine levels were slightly higher after administration of DMPA, but no changes in efavirenz levels were noted [31].

### Protease inhibitors

Four studies investigated the effects of COCs on the pharmacokinetics of protease inhibitors [38,54,55,64]. Levels of saquinavir were not affected by COC use, but atazanavir levels were slightly increased [55,64]. Co-administration with COCs resulted in darunavir and ritonavir levels comparable to those in historical controls [38,54].

Co-administration of the contraceptive patch with lopinavir/ritonavir-containing cART resulted in slightly decreased levels of both protease inhibitors compared with historical controls [37], whereas DMPA had no effect on protease inhibitor levels in women taking such regimens [39].

### Nucleoside/nucleotide reverse transcriptase inhibitors

One study found no effect of hormonal contraceptives (COCs and DMPA) on zidovudine plasma or intracellular pharmacokinetics [65].

## Chemokine receptor 5 antagonists

When maraviroc was taken with COCs, levels were similar to those seen in historical controls [60].

## Discussion

Few of the 50 reports included in this review provided relevant data that can be applied to clinical practice with certainty (Table 5). The most significant interactions with hormonal contraceptives occurred in women using cART-containing NNRTIs, particularly efavirenz. However, even in these studies, the outcomes reported were often pharmacokinetic rather than clinical, involved small populations, which limited study power, or were derived retrospectively from secondary analyses of existing cohorts.

The most important outcome for contraceptive drug interactions is method failure resulting in pregnancy, but few studies reported this outcome (Table 5). Changes in contraceptive hormone levels do not necessarily translate into reduced efficacy or increased toxicity, as levels vary greatly within and between individuals and populations [66,67]. Further, the contraceptive threshold, or minimum steroid hormone level required to maintain contraceptive effectiveness, is difficult to determine [68,69]. However, when pharmacokinetic data show no or minimal changes, clinical effects are unlikely. Ovulation is used in many drug–drug interaction studies to indicate risk of pregnancy, but ovulation is also a surrogate marker. The occurrence of ovulation does not always result in pregnancy. For example, many women ovulate during levonorgestrel implant use, yet contraceptive effectiveness remains high [70]. Additionally, no included studies evaluated true ovulation; rather, ovulation was presumed based on serum progesterone measurements alone, which can be inaccurate [70].

The most clinically significant drug–drug interactions identified in our systematic review were reported in women using efavirenz-containing cART and COCs or progestin-containing subdermal implants. Although studies show DMPA is not impacted by efavirenz use, studies of women using efavirenz and contraceptive implants reported pregnancy rates ranging from 5 to 15 per 100 woman-years, and COC users taking efavirenz had pregnancy rates ranging from 13 to 15 per 100 woman-years [24,25,35]. For COCs, because contraceptive effectiveness relies on user adherence, potential additional reductions in effectiveness from a drug interaction, if confirmed, are concerning. Conversely, studies that reported on women using contraceptives with nevirapine-containing cART were generally reassuring. None of the studies that enrolled women using a number of different hormonal contraceptives with nevirapine reported increases in pregnancy or ovulation rates [18,19,21,24,25,30,35,36,45].

The many other studies included in this review that evaluated hormonal contraceptives and antiretrovirals other than efavirenz reported no results that should change clinical practice. Antiretrovirals used in PrEP do not affect hormonal contraceptive effectiveness. Concurrent use of protease inhibitors with COCs does not alter contraceptive effectiveness despite the observed decreased ethinyl estradiol plasma levels found, as the progestin component is primarily responsible for contraceptive effectiveness. Minimal to no changes in progestin levels were reported in multiple studies of concurrent protease inhibitor and hormonal contraceptive use. Although concomitant use of a few protease inhibitors led to increased progestin levels in some studies, these changes are unlikely to impact safety given the variable doses and wide safety margin of contraceptive progestins.

Despite the small number of reports in our review, studies were also reassuring with regard to the effect of hormonal contraceptives on cART or PrEP effectiveness, or antiretroviral pharmacokinetics. Pharmacokinetic studies were limited because they either only reported antiretroviral pharmacokinetics compared with historical controls or presented data from healthy women taking single antiretrovirals.

Concurrent antiretroviral and hormonal contraceptive use also does not appear to lead to increased toxicity, though most studies were of short duration (1–28 days). Short-term pharmacokinetic studies may not accurately reflect adverse effects that may occur during use of long-term cART or PrEP with hormonal contraceptives. The only study to evaluate long-term toxicity showed little impact of concurrent use of DMPA and tenofovir on bone mineral density over 1 year [51]. Pharmacokinetic effects may also be time-dependent, further limiting the utility of short-term evaluations.

Strengths of our review included a comprehensive search strategy, systematic review of study inclusion by all authors, and dual data abstraction. Limitations are generally due to lack of studies evaluating relevant clinical outcomes. In addition, few studies are available regarding whether implants containing levonorgestrel or etonogestrel have different contraceptive effectiveness when used with efavirenz. Furthermore, with both implants, it is possible that interactions with enzyme inducers such as efavirenz are time-dependent, because hormone levels decrease over time after implantation [71]. Other data gaps are whether injectable contraceptives other than intramuscular DMPA, such as the lower-dose subcutaneous DMPA or injectable norethisterone enanthate, might be susceptible to drug interactions. Questions also remain regarding any impact of lower-dose efavirenz regimens [72] on hormonal contraceptive effectiveness, which cannot be predicted. Another limitation is that intracellular antiretroviral concentrations are likely a better predictor of clinical effectiveness than plasma levels, but pharmacokinetic studies only reported the latter. Finally, women on cART may also take other drugs that can alter liver metabolism, such as rifampin, and the combined effect of multiple enzyme-inducing medications on contraceptive hormone levels remains poorly characterized.

Our review highlights the dearth of studies designed to provide meaningful clinical data to guide contraceptive choices for HIV-positive women taking cART. Studies should be designed to report clinical outcomes such as pregnancy and HIV disease progression during long-term administration. Currently, incomplete data are being used to limit contraceptive choices for HIV-positive women. In the absence of well conducted prospective clinical trials, data from pharmacokinetic studies and secondary analyses have been used to make clinical judgments on medication effectiveness and inform contraceptive policy. For example, in October 2014 the South African authorities recommended that women using efavirenz or other enzyme-inducing drugs should not use etonogestrel implants [11]. In May 2016, the European Medicines Agency recommended that women taking hepatic enzyme inducing drugs, including efavirenz, be offered double doses of oral levonorgestrel for postcoital emergency contraception [73]. When such guidance is developed, the absolute risk of pregnancy should be considered and addressed in the guidance publications, as well as other considerations such as availability and contraceptive effectiveness of the alternatives proposed. Even if a particular contraceptive method is potentially less effective than usual in a woman using a concomitant antiretroviral, it may still be more effective than many alternative contraceptive methods [74]. Although nonhormonal methods such as copper IUDs are not affected by drug interactions, their use remains very low in many settings worldwide, and efforts to increase IUD use have had limited success [7]. If access to implants is restricted, in many settings DMPA would be the primary option available to women, virtually eliminating woman-centered decision making.

In summary, current published data do not support limiting women's access to any hormonal contraceptives. Women taking antiretrovirals for HIV treatment (in the form of cART) or prevention (in the form of PrEP) should have access to the full range of hormonal contraceptive options, and be enabled to make informed decisions about their options. Contraceptive efficacy is only one of many factors that an individual may consider when choosing a contraceptive method, and some women who are motivated to use the etonogestrel implant may wish to do so even if there is concern for decreased efficacy when used with efavirenz. National or regional restrictions on contraceptive method access, while well intentioned, supersede women's personal decisions, which may actually increase risk of unintended pregnancy if remaining contraceptive options are unacceptable or inaccessible. More well designed prospective studies are needed to examine potential drug interactions between antiretrovirals and all contraceptive methods, to better inform guidelines and counseling for the more than 16 million women living with HIV.

## Acknowledgements

This manuscript is made possible by the generous support of the American people through the United States Agency for International Development (USAID), provided to FHI 360 through cooperative agreement number AID-OAA-A-15-00045 and consolidated grant number GHA-G-00-09-00003 provided to the WHO. We also thank Drs Margaret Doherty, Marco De Avila Vitoria, and Shaffiq Essajee for their expert advice.

Roles of the authors: The WHO (MLG) initiated the idea to update this systematic review. K.N. led the conduct of the systematic review, conducted the literature searches, and coordinated review procedures and drafting of the manuscript. All authors participated in framing the study questions, developing eligibility criteria, reviewing identified studies for eligibility, abstracting study information, interpreting the data, and contributing to the writing, and editing of the manuscript. All authors reviewed and approved the final manuscript before submission.

Source of funding: K.N. has led previous systematic reviews on this topic, and authored a few of the studies included in the review. G.S.S. authored one of the studies included in the review. The WHO and USAID provided support for the writing of this systematic review and for the writing group to attend a working meeting in Geneva, Switzerland, in 2015.

Disclaimer: The contents are the responsibility of the authors and do not necessarily reflect represent the official positions of FHI360, USAID, the Centers for Disease Control and Prevention, the WHO, or other institutions with which the authors are affiliated.

### Conflicts of interest

There are no conflicts of interest.

## Supplementary Material

Supplemental Digital Content

## Figures and Tables

**Table 1:** Steroids used in currently available contraceptive methods, their liver metabolism, and effects on liver enzymes.

Contraceptive steroid	Abbreviation	Contraceptive method type (s)	Metabolism
Estrogens			
Ethinyl estradiol	EE	COC, patch, ring	Inhibits CYP2C19, CYP3A4, and CYP2B6
			Induces UGTs
			Metabolized by CYP3A4 and CYP 2C9 and UGT
Estradiol cypionate	E2C	CIC	Inhibits CYP2C19, CYP3A4, and CYP2B6
			Induces UGTs
			Metabolized by CYP3A4 and CYP 2C9 and UGT
Estradiol valerate	E2V	COC	Inhibits CYP2C19, CYP3A4, and CYP2B6
			Induces UGTs
			Metabolized by CYP3A4 and CYP 2C9 and UGT
Progestins			
Ethynodiol diacetate	EDA	COCs	Metabolized to norethindrone
Dienogest	DNG	COC	Metabolized by CYP3A4
Nomegestrol acetate	NOMAC	COC	Metabolized by CYP3A3, CYP3A4, and CYP2A6
Drospirenone	DRSP	COC	Metabolized only to a minor extent, by CYP3A4
Gestodene	GES	COC	Metabolized by CYP3A4
Norgestrel	NG	COC	Metabolized by CYP3A4
Norgestimate	NGM	COC	Metabolized by CYP3A4
Desogestrel	DSG	COC, POP	Metabolized by CYP2C9 and CYP3A4
Norethindrone, norethindrone acetate	NET	COC, POI, POP	Metabolized by CYP3A4
Norethisterone enanthate			
Levonorgestrel	LNG	COC, implant, IUD, ECP	Metabolized by CYP3A4
Norelgestromin	NGMN	Patch	Metabolized by CYP3A4
Etonogestrel	ENG	Ring, implant	Metabolized by CYP3A4
Medroxyprogesterone acetate	MPA	CIC, POI	Metabolized by CYP3A4

Data from USFDA prescribing information, and review articles summarizing published literature. CIC, combined injectable contraceptives; COC, combined oral contraceptive; CYP, cytochrome P450 isozyme; ECP, emergency contraceptive pill; IUD, intrauterine device; MPA, medroxyprogesterone acetate; POI, progestin-only injectable; POP, progestin-only pill; UGT, uridine diphosphate glucuronosyltransferase.

**Table 2:** Antiretrovirals included in the review, their liver metabolism, and effects on liver enzymes.

Generic name	Liver metabolism
NNRTIs	
Efavirenz (EFV)	Induces CYP3A4, CYP2B6, and UGTs
	Metabolized by CYP2B6 and CYP3A
Etravirine (ETR)	Induces CYP3A and inhibits CYP2C9, CYP2C19
	Metabolized by CYP3A, CYP2C9, and CYP2C19
Nevirapine (NVP)	Induces CYP3A and CYP2B6
	Metabolized by CYP3A, CYP2B6, and UGTs
Rilpivirine (RPV)	At higher doses (>3× approved 25 mg dose), induces CYP3A4
	Metabolized by CYP3A4, CYP2C19, CYP1A2, and CYP2C
Delavirdine (DLV)	Inhibits CYP3A, CYP3A4, CYP2C9, CYP2D6, and CYP2C19
	Metabolized by CYP3A and CYP2D6
Fosdevirine/GSK GSK2248761^a^	Weak inhibitor of CYP3A4 and CYP2D6
	Metabolized by CYP3A4?
NRTIs	
Zidovudine (ZDV) or azidothymidine (AZT)	Does not affect liver enzymes
	Metabolized by UGTs
Abacavir (ABC)	Does not affect liver enzymes
	Metabolized by alcohol dehydrogenase and UGTs
Tenofovir disoproxil fumarate (TDF)	Does not affect liver enzymes
	Minimal liver metabolism; mostly eliminated unchanged in urine
Emtricitabine (FTC)	Does not affect liver enzymes
	Minimal liver metabolism; mostly eliminated unchanged in urine
Didanosine (DDI)	Does not affect liver enzymes
	Minimal liver metabolism; mostly eliminated unchanged in urine
Lamivudine (3TC)	Does not affect liver enzymes
	Minimal liver metabolism; mostly eliminated unchanged in urine
Stavudine (d4T)	Does not affect liver enzymes
	Minimal liver metabolism; mostly eliminated unchanged in urine
PIs	
Ritonavir (RTV)	Induces and inhibits CYP3A
	Induces CYP1A2, CYP2C9, CYP2C19, CYP2B6, and UGTs
	Inhibits CYP2D6
	Metabolized by CYP3A, CYP2D6
Atazanavir (ATV)	Inhibits CYP3A and UGT1A1, and weak inhibitor of CYP2C8
	Mostly metabolized by CYP3A4; other pathways include UGTs
Darunavir (DRV)	Co-administered with ritonavir, inhibits CYP3A and CYP2D6
	Mostly metabolized by CYP3A
Fosamprenavir (FOS-APV)	Amprenavir, the active metabolite, induces and inhibits CYP3A4
	Metabolized by CYP3A4
Saquinavir (SQV)	Co-administered with ritonavir, inhibits CYP3A
	Metabolized by CYP3A
Tipranavir (TPV)	Co-administered with ritonavir, inhibits CYP3A and CYP2D6
	Induces CYP1A2 and CYP2C19 at steady state
	Metabolized by CYP3A
Indinavir (IDV)	Inhibits CYP3A4
	Weak inhibitor of CYP2D6
	Metabolized by CYP3A4
Nelfinavir (NFV)	Inhibits CYP3A4
	Metabolized by CYP3A, CYP2C19
CCR5 inhibitors	
Maraviroc (MVC)	Inhibits CYP2D6 at higher doses
	Metabolized by CYP3A
Vicriviroc (VCV)^a^	Does not affect liver enzymes
	Metabolized by CYP3A
Fusion inhibitors	
Enfuvirtide (ENF)	Does not affect liver enzymes
	Catabolized to constituent amino acids
Integrase inhibitors	
Dolutegravir (DTG)	Not an inducer or inhibitor of CYP enzymes
	Metabolized by UGTs and CYP3A
Elvitegravir (EVG)	Inducer of CYP2C9
	Metabolized by CYP3A4
Raltegravir (RAL)	Not an inducer or inhibitor of CYP enzymes
	Metabolized by UGTs
Pharmacokinetic enhancers	
Cobicistat (COBI)	Inhibits CYP3A, CYP2D6
	Metabolized by CYP3A

Data from prescribing information, http://medicine.iupui.edu/clinpharm/ddis/main-table, and https://aidsinfo.nih.gov/drugs. CYP, cytochrome P450 isozyme; NNRTI, nonnucleoside reverse-transcriptase inhibitor; NRTI, nucleoside or nucleotide reverse-transcriptase inhibitor; PI, protease inhibitor; UGT, uridine diphosphate glucuronosyltransferase.

^a^Development halted due to toxicity or lack of efficacy.

**Table 3:** Studies reporting clinical measures of contraceptive and/or antiretroviral effectiveness with co-administration of hormonal contraceptives and antiretrovirals.

Reference; location	Design objective (s)	Number of participants (*N*); population	Intervention/treatment	Results	Strengths; weaknesses; funding source
Scarsi *et al*. [35]Uganda	Prospective open-label pharmacokinetic studyTo assess the effect of EFV or NVP containing cART on pharmacokinetics of LNG implants in HIV+ women	Sixty HIV+ women >18 years (median age 31), medically eligible for LNG implant, not recently pregnant, and not using other potentially interacting drugs	LNG implantcART-containing NVP or EFV, or no cART	Study stopped early due to unintended pregnancies in EFV group: 3/20 (15%) women in EFV group were pregnant: two at week 48 visit and one at week 42LNG concentrations at last visit before pregnancy (week 36) were 122, 299, and 303 pg/mlZero pregnancies in either no-cART or NVP groupsNo change in CD4^+^ cell count HIV-RNA in ART groupsNo difference in adverse events	Strengths: clearly described interventions and outcomes; pregnancy confirmed by urine test; HIV+ womenWeaknesses: nonrandomized; not designed to evaluate contraceptive effectiveness; no measurement of progesteroneFunded by government
Patel *et al*. [24]Kenya	Retrospective cohortTo compare pregnancy rates among women using different contraceptives and EFV and NVP containing cart	Twenty-four thousand, five hundred and sixty HIV+ women aged 15–45 years	2% ENG implant 5% LNG implant 17% DMPA 3% COCs/oral contraceptives 3% IUDs/permanent49% NVP-containing cART 14% EFV-containing 4% LPV/r-containing 32% no cART	3337 incident pregnancies: overall pregnancy rate of 8.9/100 woman-yearsUnadjusted pregnancy rates by method and antiretroviral type (per 100 woman-years)COCs/oral contraceptives NVP 10.9 EFV 15.4 LPV 15.4 no cART 11.1DMPA NVP 8.4 EFV 9.4 LPV 7.2 no cART 9.8ENG implant NVP 2.3 EFV 5.5 LPV 1.3 no cART 3.4LNG implant NVP 1.9 EFV 7.1 LPV 0 pregnancies no cART 3.3	Strengths: very large sample size; HIV+; good data on cART use; comparisons made to no contraception; able to separate implant type; pregnancy as main outcomeWeaknesses: retrospective; self-reported pregnancy; high pregnancy rates even with no cART; self-reported contraceptive use; oral contraceptive use included progestin-only pills; no information on contraceptive method insertion/timing; different visit schedule for those on cART and those not on cartFunded by government
Pyra *et al*. [25]Kenya, Uganda	Secondary analysis of HIV prevention trialsTo understand the effect of cART on contraceptive effectiveness	Five thousand, one hundred and fifty-three HIV+ women <50 years; not sterilized and not using IUDs	9% implants 40% injectables 14% oral contraceptives31% used cART: 23% NVP-containing regimen 5% EFV-containing	Pregnancy rates (per 100 woman-years)Implant no cART 1.4 NVP 0 EFV 6.0Injectable no cART 5.3 NVP 3.3 EFV 3.8oral contraceptives no cART 11.0 NVP 6.4 EFV 12.9	Strengths: large sample size; prospective data collection; pregnancy diagnosed by urine pregnancy testWeaknesses: self-reported contraceptive and cART use; no information on contraceptive method timing or implant type; few women using EFV-containing regimensFunded by government
Callahan *et al*. [41]Kenya, South Africa	Secondary analysis of HIV prevention trialTo describe contraceptive effectiveness and predictors of pregnancy; and relationship between TDF/FTC and contraceptive effectiveness	Two thousand, one hundred and twenty sexually active women at risk of HIV enrolled in PrEP trial; median age 23; median BMI 23; use of effective contraceptive required at enrollment	∼50% not using any method or using condoms alone at screening At enrollment: 55% injectables 43% COCs 2% implants, IUDs, or sterilizationTDF/FTC or placebo	Pregnancy rates (per 100 woman-years)Overall 9.6 TDF/FTC 11.4 placebo 7.7Implant 0Injectable: TDF/FTC 2.2 placebo 1.1COCs: TDF/FTC 34.9 placebo 27.9 new users 35.1 prior users 20.7TDF/FTC had no effect on contraceptive effectiveness of COCs in final modelWomen on TDF/FTC and COCs had more nausea or vomiting (8.1%) vs. COC + placebo group (1.4%)	Strengths: large cohort over long period; pregnancy confirmed by urine tests; contraceptive methods dispensed at study clinic; TDF/FTC use verified by blood levels; low loss to follow-upWeaknesses: poor adherence to TDF/FTC; self-reported COC useFunded by government
Whiteman *et al*. [43]Russia	Prospective cohortTo examine the associations between hormonal contraceptive use and HIV progression and cART effectiveness	Seven hundred and nine sexually active HIV+ women; age 16–45 years; not pregnant or breast-feeding; no hysterectomy, infertility, or recent hormonal contraceptive or IUD use	At enrollment: 183 COCs 87 DMPA 156 nonhormonal methodscART-containing either PI or NNRTI	545 not on cART 161 on cARTThree women discontinued due to unrecognized pregnancy at enrollmentFive pregnancies during study but contraceptive method or cART use not reportedNo significant change in CD4^+^ cell count or viral suppression between COC or DMPA and nonhormonal usersTwo deaths; no report of which contraceptive method used	Strengths: cART and contraceptive use verified; pregnancy by urine test or medical records; death verified by recordsWeaknesses: very high loss to follow-up; unclear if women becoming pregnant were using contraceptives; limited information on cART regimenFunded by government
Song *et al*. [42]USA	Randomized; double-blind; placebo-controlled; crossover studyTo examine the effect of DTG on pharmacokinetics and pharmacodynamics of EE and NGMN in COC users	Sixteen healthy women; age 18–40; mean age 31 years; 94% white; BMI 19–30 (mean 24.7); normal liver function; women had to use a second nonhormonal contraceptive	COC-containing NGMDTG 50 mg or placebo twice daily for 11 days	Fifteen women completed studySerum pregnancy test 7–14 days after last dose; zero pregnanciesNo difference in LH, FSHProgesterone data not reportedNo participants discontinued due to adverse events; no grade 3 or 4 or serious adverse events	Strengths: randomized; clearly described population and methods; progesterone measured several times during cycleWeaknesses: small sample size; healthy women; single antiretroviral; single cycle; ovulation data not reported and unclear from figureFunded by industry
Kasonde *et al*. [51]Botswana	Secondary analysis of RCTTo investigate the effect of TDF and the interaction of TDF and hormonal contraception on BMD among HIV-uninfected African men and women	One hundred and fourteen sexually active women at risk of HIV, from HIV prevention trial; 18–39 years; nonpregnant and nonbreast-feeding	Injectable or implant oral contraceptivesTDF TDF/FTC placebo	Data not separated for women vs. menBone mineral density with DEXA at distal and ultradistal forearm; lumbar spine; hip3/114 (2.6%) had a low baseline bone mineral density;Changes in bone mineral density for women on either oral or injectable vs. no contraception not significant except for a positive effect of oral contraceptives on spine bone mineral density for women on TDF/FTC	Strengths: used DEXA to measure bone mineral densityWeaknesses: some results not separated by gender or HIV status; no mention of pregnancy or lactation or other medication use; few data on contraceptive use; low adherence to TDF/FTC; unclear if injectable group also included implant usersFunded by government
Luque *et al*. [39]USA	Open-label; multicenter; nonrandomized; steady-state pharmacokinetic studyTo assess the effect of LPV/r on DMPA pharmacokinetics and vice versa; and to assess safety and tolerance of DMPA given concurrent with LPV/r	Twenty nonpregnant; premenopausal HIV+ women; on stable LPV/r for at least 14 days; no DMPA within 180 daysMedian BMI 28	DMPAcART-containing LPV/r	No pregnanciesProgesterone >5 ng/ml considered presumptive ovulation; zero ovulations notedNo serious adverse events; one grade 3 adverse event (prolonged bleeding).No changes in CD4^+^ cell count or HIV RNA through week 8At week 12–3/24 women in LPV/r group had detectable HIV RNA; two due to antiretroviral noncompliance	Strengths: clearly described population and methods; HIV+ women; assessed ovulation at several time pointsWeaknesses: small sample size; short durationFunded by government
Todd *et al*. [59]Kenya	Secondary analysis of PrEP HIV prevention trialTo examine PK of LNG with concurrent use of TDF-FTC as PrEP	Twenty-nine sexually active women at risk of HIV, who elected to received LNG implant; ages 18–35TDF/FTC group: *N* = 17Placebo group: *N* = 12Mean BMI 22.6	LNG implantTDF/FTC or placebo	Follow-up 36 weeksNo pregnancies and one implant discontinuation at 7 months, with reason for discontinuation not recorded	Strengths: TDF levels measured to assess for adherenceWeaknesses: Small sample size; percentage retention not statedFunded by government
Heffron *et al*. [50]Kenya, Uganda	Secondary analysis of PrEP RCTTo evaluate TDF/FTC and TDF efficacy among women using DMPA compared with nonhormonal users	One thousand, seven hundred and eighty-five women at risk of HIV; median age 33 years	At enrollment: 486 DMPA usersIn follow-up: additional 415 DMPA usersTDF/FTC, TDF, or placebo	Efficacy of PrEP not different for women using DMPA compared with women using no hormonal contraceptionAmong DMPA users: efficacy 64.7% (PrEP vs. placebo) Among nonhormonal users: efficacy 75.5% (PrEP vs. placebo)*P* interaction = 0.65No data about pregnancy reported	Strengths: large sample size; high adherenceWeaknesses: secondary analysis; self-reported contraceptive use; adjustment for unprotected sex but unclear whether or how condom use was collectedFunded by government
Day *et al*. [44]Kenya	Prospective cohortTo test the hypothesis that DMPA would be associated with increased detection of HIV-1 RNA in women initiating and continuing cart	One hundred and two HIV+ women starting cART; median age 36; median CD4^+^ cell count 122 cells/μl	At baseline: 18 (18%) DMPA 5 (5%) implants; 5 (5%) oral contraceptivescART-containing ZDV; d4T; 3TC; and NVP	Seventy two completed ≥33 months follow-upDMPA did not increase plasma HIV RNA	Strengths: long follow-up; adjusted for antiretroviral adherence and CD4^+^ cell countWeaknesses: self-reported contraceptive and antiretroviral use; large loss to follow-up; 14% changed cART regimen; small number of women using DMPAFunded by government
Atrio *et al*. [16], Atrio *et al*. [56], Dubois *et al*. [17]USA	Nonrandomized clinical trialTo evaluate the effect of protease inhibitors on cervical mucus of POP users	Thirty-five HIV+ women, age 18–44 years; no changes in medications; no recent hormonal contraceptives; no immunocompromise; no liver or renal disease; normal ovulation; BMI <40; >30 days postpartum	POPs containing NETIn PI group: 11 taking cART containing ATV (10/11 on ATV/r); 3 DRV/r; 2 LPV/rIn control group: four women not taking cART; 13 taking combinations including ETR, RPV, TDF, FTC, and RAL	Baseline mucus scores similarCervical mucus scores in PI and non-PI groups similar after POPs: median score 3.5 for PI group and four for controls score <10 (unfavorable to sperm penetration): 81% of study group; 60% of comparison group	Strengths: prospective design; blinded assessmentsWeaknesses: no baseline of periovulatory mucus for all women; small sample size; nonrandomized; cART use self-reported; results not separated by antiretroviralFunded by government
Murnane *et al*. [40]Kenya, Uganda	Secondary analysis of PrEP HIV prevention trialTo assess the impact of TDF and TDF/FTC on hormonal contraceptive effectiveness	One thousand, seven hundred and eighty-five sexually active women at risk of HIV, enrolled in HIV prevention trial; median age 33	At enrollment: 27% injectablesDuring follow-up: 14% initiated oral contraceptives 20% injectables 6% implantsTDF/FTC; TDF; or placebo	Two hundred and eighty eight pregnancies in 267 women (179 TDF or TDF/FTC and 88 placebo); no difference across armsPregnancy rates per 100 women-yearsOral contraceptives TDF or TDF/FTC 17.7 placebo 10.0; no significant differenceInjectablesTDF or TDF/FTC 5.1 placebo 5.3Implants <1% per year in both arms	Strengths: large sample size; verified contraceptive use; good adherence to study product;Weaknesses: nonrandomized; oral contraceptive, implant, and injectable type not specified; contraceptive use and adherence self-reportedFunded by government
Perry *et al*. [36]Switzerland	Retrospective cohortTo evaluate risk of pregnancy in implant users using cART	Five hundred and seventy HIV+ women who had LNG implant	LNG implants347 (61%) using cART at implant insertion: 208 on NVP-containing regimens; 121 on EFV-containing regimens; 18 on LPV/r-containing regimens	Sixteen pregnancies in 570 women15/121 (12.4%) of women on EFV became pregnant; mean time between implant insertion and pregnancy was 16.4 monthsNo women conceived while using NVP or LPV/r, one pregnancy occurred before cART was startedAge, condom use, inserting provider, CD4^+^ cell count had no association with pregnancy	Strengths: large sample size; HIV+; verified contraceptive use, implant insertion date knownWeaknesses: retrospective study; self-reported cART use; 3/16 women who became pregnant had received antituberculosis treatment; no BMI informationFunded by hospital
Vieira *et al*. [34]Brazil	Prospective cohort with pharmacokinetic analysisTo evaluate the effects of EFV-containing or LPV/r-containing cART on ENG implant pharmacokinetics and to determine the impact of cART on luteal activity	Forty five HIV+ women with regular menstrual cycles, BMI 18–30; excluded women with recent pregnancy or hormonal contraceptive use, acute infections or other opportunistic illnesses, drug or alcohol addiction, use of other potentially interacting drug, chronic diarrhea or malabsorption or noncompliance with cART	ENG implantLPV/r-containing or EFV-containing cART, or no cART	Progesterone measured every 2 weeks; >4.7 ng/ml considered presumptive ovulation; progesterone >3 ng/ml considered luteal activity2.8% of the P samples in EFV group had presumptive ovulation; and 5% had luteal activityNo women in LPV/r or no cART groups had evidence of any luteal activityVL <50 copies/ml in LPV/r and EFV groups	Strengths: clearly described population and methods; frequent progesterone measurementsWeaknesses: Small sample size; nonrandomizedFunded by government
Hubacher *et al*. [45]Kenya	Prospective cohortTo examine how concurrent use of hormonal contraceptive implants and cART might lessen the effectiveness of both medications	Ninty-three sexually active HIV+ nonpregnant women, age 18–44 years; CD4^+^ cell count ≥ 200 cells/μl; without recent hormonal contraceptive or rifampin use, desire for pregnancy, or contraindications to implant use	LNG implant or nonhormonal contraceptionNVP-containing cart	LNG implant users (60 recruited; 48 analyzed) matched to women not using hormonal contraception (36 recruited; 33 analyzed)CD4^+^ cell counts for both groups rose slightly but did not differ between groupsNo participants died; six participants (two implant users, four controls) diagnosed with opportunistic infectionsZero pregnancies in implant users	Strengths: large sample size; implant inserted at study siteWeaknesses: method of pregnancy ascertainment not stated; observational study; no non-cART users; six women (10%) of implant group had implant removed within 12 months; cART self-reportedFunded by government
Landolt *et al*. [15,19]Thailand	Prospective; open-label; nonrandomized steady-state clinical trialTo assess risk of ovulation and safety in women taking COCs with cart	Forty-nine HIV+ nonpregnant, nonlactating women; 18–45 years, with regular menses, on EFV-containing or NVP-containing cART; nonsmoking, no recent injectable contraceptive use, no contraindications to COCsFourteen HIV− controls	COC containing DSG for two cyclesNVP-containing or EFV-containing cART	Forty-eight completed study, 15 discontinued, including 13 due to protocol adherence issuesOvulation by serum progesterone:NVP group: All women had progesterone <1.0 ng/mlEFV group: three women had progesterone >3.0 ng/mlMore women in EFV group reported adverse events than NVP group	Strengths: prospective clinical trial; HIV+ womenWeaknesses: nonrandomized; small sample size; single progesterone measurement; no adherence information; high dropout rateFunded by government
Nanda *et al*. [18]Uganda, South Africa	Prospective open-label nonrandomized clinical trialTo compare ovulation and pregnancy rates between two groups of women: those taking COCs concurrently with NVP-containing cART and those taking COCs alone	Four hundred and two sexually active HIV+ women 18–35 years, with regular menses and no contraindications to COC use; median age 29 and median CD4^+^ cell count 486 cells/μl	COC-containing NGcART group included women on NVP-containing cART; *n* = 196Control group included women not yet eligible for cART; *n* = 206	Ovulation by serum progesterone (>3 ng/ml)cART group: 43/168 (26%) ovulated in cycle 1; 30/163 (18%) in cycle 2; 18/163 (11%) in both cyclesNon-cART group: 26/168 (15%) ovulated in cycle 1; 31/165 (19%) in cycle 2; and 20/165 (12%) in both cyclesNo significant difference in ovulation rates between groupsPregnancy rates (per 100 woman-years): 10. in cART group and 10.1 in non-cART groupAdverse events similar; five serious adverse events, all in non-cART group	Strengths: prospective clinical trial; COCs and antiretrovirals at steady state; multiple progesterone measurements; large sample size; HIV+ women; information on COC adherenceWeaknesses: nonrandomized, self-reported cART and COC adherence; no pharmacokinetic measuresFunded by government
Crauwels *et al*. [27]UK	Open-label, three period pharmacokinetic studyTo evaluate the effect of RPV on COC pharmacokinetics and vice versa, and assess effects on sex hormones and safety of co-administration	Eighteen healthy nonsmoking women, 18–45 years; BMI 18–30 (median 24.6); 67% white; excluded pregnant, breast-feeding, or menopausal women, those with history of drug/alcohol abuse, skin disease, or any significant medical problems; use of concomitant medication	COC-containing NETIn third cycle; RPV 25 mg daily days 1–15	Thirteen completed trialProgesterone; LH; and FSH on day 1 and 14; 0 ovulations; no effect on FSH; LHNo difference in adverse events	Strengths: clearly described population and methods; directly observed therapyWeaknesses: healthy women; short-term dosing; single antiretroviral; high discontinuation rateFunded by industry
Polis *et al*. [46]Uganda	Retrospective cohortTo assess the effect of injectable contraceptive use on cART effectiveness and adherence to cart	Four hundred and eighteen pregnant and nonpregnant sexually active HIV+ women initiating cART, without tuberculosis, with information on baseline viral load	51/418 (12%) used unspecified injectables at baselinecART (not specified)	Failure defined as failure to achieve virologic suppression at 12 months, switch to second-line therapy, or death within 12 months of cART initiationNo difference in treatment failure at 12 months between injectable users and nonusers (11 vs. 12%)Injectables not associated with cART failure in sensitivity analysis restricted to women with complete information who never used pills or implantsNo differences in cART adherence at 6 and 12 months for injectable users and nonusers	Strengths: large sample sizeWeaknesses: retrospective; observational database analysis; self-reported contraceptive use; inconsistent injectable use over time; type of injectable and cART not specifiedFunded by government
Carten *et al*. [52]USA	Open-label two period pharmacokinetic studyTo determine the effect of EFV on the pharmacokinetics of LNG EC and vice versa, and assess safety	Twenty-four healthy women; 18–45; normal BMI (mean BMI 27) with no recent use of hormonal contraceptives or other interacting medications; women were either sterilized or used two nonhormonal contraceptive methods	LNG ECPs (0.75 mg) at 0 and 12 h on days 0 and 17EFV 600 mg 72 h after day 0, for 14 days	Twenty-one women completed studyFollow-up pregnancy test at visit 3 (study day not specified)Pregnancy test results not givenNo grade 3 or 4 treatment-related toxicities.	Strengths: clearly described population and methodsWeaknesses: small sample size; healthy women; single antiretroviral; ovulation not tested; single follow-up pregnancy test but timing and results not givenFunded by industry
Piscitelli *et al*. [29]UK	Randomized crossover pharmacokinetic studyTo examine if GSK2248761 (fosdevirine) interacts with CYP450 substrates, including COCs	Ten healthy women, without hepatitis and not taking any medications	COC containing DRSPGSK228761 200 mg or placebo days 1–11	No differences in LH/FSHNo serious adverse events or treatment due to adverse events, and no significant laboratory abnormalities	Strengths: randomizedWeaknesses: short term administration; healthy women; single antiretroviral; small sample size; very few study details provided; trials of fosdevirine on hold due to other safety concerns; study terminated early for unknown reasonsFunded by industry
Schwartz *et al*. [21]South Africa	Prospective cohortTo determine the incidence of unplanned pregnancies in HIV+ women on cART; to assess contraceptive use and associations with unplanned pregnancy	Eight hundred and fifty HIV+ women; ages 18–35; on/starting cART; not pregnant, recently pregnant, or breast-feeding; no known infertility	243 (29%) using HC: Injectables (DMPA + NET-EN; 192); COCs (46); implants (type not stated 4); IUD (1)Multiple antiretrovirals: 52% NVP-containing regimens; 42% EFV-containing	One hundred and seventy pregnancies in 161 women; 105 (62%) unplanned (incidence rate: 16.1/100 woman-yearsNine of 105 unplanned pregnancies were potentially hormonal contraceptive failures; seven on NVP and one on EFV; incidence of unplanned pregnancy 4.4 per 100 woman-yearsOne failure not related to adherence in COC user (5.8/100 woman-years)Seven injectable failures (two DMPA; five NET-EN; (incidence rate 4.2/100 woman-years); 5/7 in last 2 weeks of injection cycle	Strengths: pregnancy by urine hCG; cART confirmed by pharmacy records; contraceptive failures confirmed through records reviewWeaknesses: observational study; contraceptive use self-reported; reported only at baseline; did not report which HC failures were using which antiretroviralFunded by government
Kreitchmann *et al*. [33]Brazil	Prospective cohortTo evaluate the safety and efficacy of ENG implants among HIV+ women	Seventy nine HIV+ women with comorbidities and poor adherence to other contraceptive methods; mean age 29; mean weight 59 kg (range 42–104)	ENG implantAt baseline: 47 used cART; nine began cART during follow-up (PI containing-regimen 31; NNRTI-containing 25)	Women followed up every 6 months over 3 years and 0 pregnancies notedFour women had elevated liver enzymes: all coinfected with hepatitis CENG implant removed in five women: two had tubal ligation; one hysterectomy; two because of excessive bleedingMenstrual irregularity most common adverse event; two unrelated deaths: one of AIDS, and one of cardiac arrest (baseline cardiomyopathy)	Strengths: verified contraceptive use; prospective study; HIV+ womenWeaknesses: self-reported pregnancy; did not specify cART typeFunding source not specified
Johnson *et al*. [47]USA	Retrospective cohortTo examine how use of hormonal contraceptives affects response to cART	One hundred and seven HIV+ adolescent women reporting consistent cART ; median age 17 years	Seventy-two percent oral contraceptivesTwenty eight percent DMPAcART regimens included ZDV and ZDV/3TC	No difference in CD4^+^ cell counts over timeViral load decreased over time in hormonal users and nonusers; but an interaction was noted: decrease in viral load was slightly slower (1.2 × 10^−3^; 95% CI: 6.2 × 10^−5^ to 2.4 × 10^−3^ copies/ml log viral load per day; *P* = 0.03) among hormonal contraceptive users	Strengths: HIV+ womenWeaknesses: retrospective; changes in viral load of questionable clinical significance; contraceptive use self-reported; not separate type of contraceptivesFunded by government
Stuart *et al*. [30]Malawi	Prospective nonrandomized clinical trialTo assess the feasibility of measuring anovulation in a pharmacokinetic study of COCs and antiretrovirals	Nine women ages 21–35 (3/group) with similar age and BMI: group 1 included HIV+ women on cART; group 2 included HIV+ women not on cART; and group 3 included HIV− women	COC with NGNVP-containing cART or no cART	Ovulation by serum progesterone (>3.0 ng/ml) on day 14; 0 ovulations	Strengths: Clearly described population and methods; valid assays; included HIV+ womenWeaknesses: very small sample size; nonrandomized; progesterone measured only onceFunding source not reported
Sevinsky *et al*. [26]USA	Open-label 3-period pharmacokinetic studyTo examine effect of EFV on pharmacokinetics of EE and NGMN and vice versa	Twenty-eight healthy women; 18–45 years (median 26); BMI 20–32 (median 25); on COCs for at least 2 months and no baseline safety issues or breakthrough bleeding	COC-containing NGMEFV 600 mg daily for 14 days during third cycle	Nineteen women completed studyPregnancy test day 108; results not reportedProgesterone levels similar and all <1.25 ng/mlNo discontinuations for adverse events; three severe adverse events: headache; anhedonia; and depression; one serious adverse event – suicide attempt after treatment in a woman with prior undisclosed depression	Strengths: clearly described population and methodsWeaknesses: small sample size; single progesterone measurement per cycle; pregnancy testing results not reported; healthy women; single antiretroviral; high discontinuation rateFunded by industry
Vogler *et al*. [37]USA	Open-label; 4-week; nonrandomized; comparative clinical trialTo evaluate pharmacokinetics interactions between LPV/r and contraceptive patch and COCs	Thirty two nonpregnant; premenopausal HIV+ women >13 years either on stable LPV/r-containing regimens for at least 14 days (*n* = 8) or not on cART or taking NRTIs only (*n* = 24); nonsmoking; median weight 72 kg; no recent use of injectables or COCs; <198 lb; not taking enzyme inducers	EE/NGMN contraceptive patchSingle-dose COC containing NETLPV/r (400 mg/100 mg twice a day) or no cART/NRTIs only	Zero ovulations by serum progesteroneHIV-1 RNA and CD4^+^ cell counts measured at 30–45 days prior to entry; at entry and 4 weeksMedian CD4^+^ ↑15% (LPV group) with maintenance of viral suppressionTreatment arm: single possibly related grade-3 adverse event (generalized aches and pains); 3 patients with 7 w/grade 1–2 adverse events; 14 control pts w/grade 1–2 adverse events; No significant changes in weight; chemical or lab values	Strengths: clearly described population and methods; HIV+Weaknesses: study stopped due to slow accrual; small sample size; single progesterone measurement; single dose COC; high loss to follow-up; not randomisedFunded by government
Myer *et al*. [20]Cote d’Ivoire, Kenya, Rwanda, South Africa, Uganda, Zambia	Secondary analysis of MTCT-Plus Initiative cohortTo examine whether improved health from cART affects pregnancy rates	Four thousand, five hundred and thirty-one HIV+ women who had received PMTCT services; median age = 27	At baseline 1; 755 (39%) reported contraceptive use: Injectables (15%) oral contraceptives (4%) IUDs (1%)Among women who started cART; 90% used NVP-containing regimens	Five hundred and eighty-nine pregnancies in 4531 women (7.8/100 woman-years)Higher pregnancy rate in women taking cART (9.0/100 woman-years) compared with women not on cART (6.5/100 woman-years)In injectable users pregnancy rates (per 100 woman-years) 1.1 before cART and 2.0 after cartIn oral contraceptive users pregnancy rates (per 100 woman-years) 3.1 before cART and 5.4 after cart	Strengths: large sample size; prospective data collection; long follow-up; HIV+Weaknesses: unclear whether women were actually using contraceptives when pregnancy occurred; study not designed to look at pregnancy; self-reported pregnancy and contraceptive useFunded by private foundation
Schöller-Gyüre *et al*. [28]USA	Open-label three period pharmacokinetic studyTo assess the effect of ETR on COC pharmacokinetics	Twenty-four healthy women; 18–45 years (median 24); BMI 18–30; 97% white; nonsmoking; not pregnant or breast-feeding; no contraindications to hormonal contraceptives; not taking enzyme inducers	COC-containing NETIn cycle 3; 200 mg ETR twice daily from day 1–15	Serum progesterone; LH; FSH on days 1 and 14 of cycles 2 and 3Zero ovulations; no difference in LH; FSHNine adverse events led to discontinuation: seven grade 2 rashes; one grade 2 pyrexia	Strengths: clearly described population and methods; valid assaysWeaknesses: healthy women; small sample size; few progesterone measurements; single antiretroviralFunded by industry
Nanda *et al*. [32]Brazil	Nonrandomized, controlled, open-label pharmacokinetic studyTo evaluate the effect of EFV-containing cART on pharmacokinetics of MPA, and to evaluate suppression of ovulation and bleeding patterns	Thirty-three HIV+ women aged 19–40 years; with regular menstrual cycles and BMI 18–30 kg/m^2^; not recently pregnant or breast-feeding	DMPAEFV-containing cART vs. no cart	Progesterone measured every 2 weeks; >3 ng/ml considered evidence of ovulation: one ovulation in non-cART group at 12 weeksNo differences in bleeding patterns between groupsNo serious adverse events	Strengths: Clearly described population and methods; HIV+ women; frequent progesterone measurementsWeaknesses: small sample size, no progesterone levels beyond 12 weeksFunded by government
Sekar *et al*. [38]Belgium	Randomized crossover pharmacokinetic studyTo investigate the effect of DRV/r on COC pharmacokinetics and to examine safety	Twenty-two nonsmoking healthy women, 18–45 years; BMI 18–30; using a second nonhormonal contraceptive method	COC-containing NETDRV/r 600/100 mg twice daily days 1–14 or no treatment	Progesterone; LH; FSH on days 1 and 14 of each cycleNo significant changes in LH or FSH with co-administrationNo serious adverse events or grade 3 or 4 adverse events; five women discontinued due to grade 2 cutaneous reactions with combined treatment	Strengths: clearly described population and methods; randomizedWeaknesses: 17-OH progesterone levels presented instead of progesterone levels; small sample size; healthy women; single antiretroviral; high discontinuation rateFunded by industry
Cohn *et al*., Watts *et al*. [14,31]USA	Nonrandomized open-label pharmacokinetic studyTo evaluate the effect of various antiretrovirals on pharmacokinetics of MPA and vice versa; and to determine effects on suppression of ovulation and adverse events	Seventy-two HIV+ nonpregnant; premenopausal women, 22–46 years (median 35); median weight 71 kg; with no recent potentially interacting drugs; women required to use second nonhormonal method of contraception	DMPASixteen on no PI or NNRTI (control) 21 on nelfinavir and NRTIs 17 on EFV and NRTIs 16 on NVP and NRTIs	Zero pregnanciesProgesterone every 2 weeks; >5 ng/ml considered presumed ovulation; zero ovulationsNo changes in median CD4^+^ cell count or proportion with viral load <400 copies/mlNo grade 3 or 4 related adverse events.	Strengths: Clearly described population and methods; HIV+ women; frequent progesterone measurementsWeaknesses: nonrandomized; small sample size; progesterone only measure up to 12 weeksFunded by government
Danel *et al*. [22]Cote d’Ivoire	Prospective cohortTo evaluate the efficacy and tolerance of ZDV; 3TC; and EFV in West African women and men	Five hundred and forty-eight HIV+ women <18 years (median age 23), naive to cART, CD4^+^ cell count 150–350 cells/ml	Approx. 80 reported using contraceptives: 65% ‘intramuscular progesterone’ 35% COCsEFV-containing cart	Seven pregnancies; incidence 2.6/100 person-years (95% CI 0.67–4.51)	Strengths: HIV+ women; large sample size; prospectiveWeaknesses: study not designed to measure pregnancy; unclear whether women were actually using contraceptives when conception occurred; did not separate injectables from oral contraceptivesFunded by government
Chu *et al*. [48]USA	Retrospective cohortTo determine the effects of hormonal contraceptives on response to cart	Seventy-seven hormonal contraceptive users matched with 77 nonusers from the Women's Interagency HIV Study; all women took cART	Seventy-seven hormonal contraceptive users: 64% COC 27% DMPA 4% LNG implant 4% COCs and DMPASeventy-seven women not using hormonal contraceptivescART containing NRTIs + PI or NNRTI; or NRTIs alone	By fourth visit; 65% stopped hormonal contraceptivesNo significant difference in CD4^+^ cell count and HIV viral load by hormone use after antiretroviral initiation except in viral load at the third visit after initiationTime-dependent hormonal contraceptive use not associated with changes in CD4^+^ cell count or undetectable viral load after cART initiation	Strengths: matched comparison group, HIV+ womenWeaknesses: retrospective; low overall use of hormonal methods contraceptive information obtained retrospectively and mainly at baseline (before cART); did not separate COCs from progestin-only methods; high method discontinuation rateFunded by government
Clark and Theall [23]USA	Retrospective cohortTo determine the frequency of oral contraceptive failure among HIV+ women on cART	Two thousand and fifty-three women	86 (4.2%) taking oral contraceptivescART containing various antiretrovirals	Forty one women were pregnant with records showing hormonal contraceptive during the same 6-month periodEleven of 41 women apparently conceived while using hormonal contraceptives (DMPA = 1 or oral contraceptives = 10)Women on NFV-containing regimens more likely to experience oral contraceptive failure	Strengths: large sample size, HIV^+^ womenWeaknesses: retrospective chart review; unclear whether women actually taking contraceptives at time of pregnancy; difficult to interpret findings; no data on cART use or adherence; no details about pregnancy; very small numbers of cases; timing of HC and cART use not clearFunding source not specified
Frohlich *et al*. [64]Germany	Open-label single period pharmacokinetic studyTo investigate the influence of COCs on SQV pharmacokinetic and to assess the potential contribution of CYP3A4 and P-gp	Eight healthy nonsmoking nonpregnant women with regular menses; mean age 24 years and mean BMI 21; not using any potentially interacting drugs	COC containing GES days 4–25	Estradiol; progesterone; LH; FSH on days 1 and 22COC use resulted in decreased plasma estradiol levels; progesterone; FSH; and LH; and increased SHBG	Strengths: Clearly described population and methodsWeaknesses: did not evaluate the effect of SQV use on ovarian suppression; not randomized; very small sample size; short course of COCs; healthy women; single antiretroviral only given twiceFunded by government
Cejtin *et al*. [49]USA	Retrospective cohortTo compare HIV-1-RNA and CD4^+^ cell counts from users and nonusers of hormonal contraception	Premenopausal HIV+ women <50 years of age	One hundred and seventy-seven users of hormonal contraception: 87 oral contraceptives 77 DMPA 13 LNG implantOne thousand, five hundred and forty-four nonusers of hormones40.7% of users and 32.9% of nonusers on cART; most on monotherapy	Hormonal contraceptive use not associated with significant changes in viral load or CD4^+^ cell count	Strengths: Some longitudinal data and large study sizeWeaknesses: no disaggregation by type of cART or contraceptive method; difficult to interpret findings; self-reported contraceptive useFunded by government
Mildvan *et al*. [53]USA	Open-label, single dose, two period pharmacokinetic studyTo determine the effects of NVP on COC pharmacokinetics and vice versa	Fourteen HIV+ nonpregnant, nonlactating, nonsmoking women; age 18–65 (mean age 37); viral load <400; CD4^+^ cell count >100; normal renal and hepatic function; no RTV or DLV use	Single dose of COC containing NET on cycle days 1 and 30NVP 200-mg daily on days 2–15; then 200-mg twice daily days 16–29; single dose on day 30cART regimens included IDV; NFV; SQV/RTV	Ten women completed the studyPregnancy test on day 30HIV RNA and T cells measured at screening, day 0, day 30No change in HIV RNA concentration or CD4^+^ cell count on day 30 compared with baselineEight of 14 had at least one adverse event; no adverse event considered related to COC	Strengths: HIV+ women, well described populationWeaknesses: small study; only single dose COC; NVP added to current cART regiment; included postmenopausal womenFunded by industry

Abbreviations for antiretrovirals and contraceptive steroids defined in Tables 1 and 2. cART, combination antiretroviral therapy; COC, combined oral contraceptive; DEXA, dual energy x-ray absorptiometry; DMPA, depot medroxyprogesterone acetate; ECP, emergency contraceptive pill; FSH, follicle stimulation hormone; HC, hormonal contraceptive; LH, luteinizing hormone; MPA, medroxyprogesterone acetate; NET-EN, norethisterone enanthate; POP, progestin-only pill; PrEP, preexposure prophylaxis; SHBG, sex hormone binding globulin.

**Table 4:** Studies reporting pharmacokinetic outcomes with co-administration of hormonal contraceptives and antiretrovirals.

Reference; location	Design objective (s)	Number of participants (*N*); Population	Intervention/treatment	Results	Strengths, weaknesses; funding
Scarsi *et al*. [35]Uganda	Prospective open-label pharmacokinetic studyTo assess the effect of EFV-containing or NVP-containing cART on pharmacokinetics of LNG implants in women living with HIV	Sixty HIV+ women >18 years (median age 31), medically eligible for LNG implant, not recently pregnant, and not using other potentially interacting drugs	LNG implantcART-containing NVP or EFV or no cART	Primary endpoint: LNG levels at 24 weeks (compared with no cART group) EFV group ↓47% NVP group ↑35%Secondary: LNG levels at 48 weeks EFV group ↓57% NVP group ↑14%EFV and NVP levels not affected by LNG	Strengths: clearly described interventions and outcomes; valid assays; HIV+ womenWeaknesses: nonrandomized; open label; LNG levels much higher than previously seen in other studiesFunded by government
Song *et al*. [42]USA	Randomized; double-blind; placebo-controlled; crossover studyTo examine the effect of DTG on pharmacokinetic and pharmacodynamic of EE and NGMN in COC users	Sixteen healthy women; age 18–40; mean age 31 years; 94% white; BMI 19–30 (mean 24.7); normal liver function; women had to use a second nonhormonal contraceptive	COC-containing NGMDTG 50 mg or placebo twice daily for 11 days	EE levels unchanged NGMN AUC; *C*_min_; *C*_max_ unchangedDTG levels similar to historical controls	Strengths: randomized; clearly described methods; valid assaysWeaknesses: healthy women only; single cycleFunded by industry
Luque *et al*. [39]USA	Open-label nonrandomized pharmacokinetic studyTo assess the effect of LPV/r on DMPA pharmacokinetics and vice versa; and to assess safety and tolerability	Twenty-four nonpregnant premenopausal HIV+ women with no recent DMPA; median BMI 28; HIV RNA <400 copies/ml	DMPA; LPV/r containing cARTcART-containing LPV/r	MPA levels compared with historical controls: AUC↑ 46% *C*_max_ ↑66%No changes in LPV or RTV levels after DMPA	Strengths: clearly described population and methods; valid assays; HIV+ womenWeaknesses: small sample size; historical controls; levels only assessed through 12 weeksFunded by government
Todd *et al*. [59]Kenya	Secondary analysis of PrEP HIV prevention trialTo examine pharmacokinetic of LNG with concurrent use of TDF/FTC as PrEP	Twenty-nine healthy women who elected to received LNG implant; ages 18–35TDF/FTC group: *N* = 17Placebo group: *N* = 12Mean BMI 22.6	LNG implantTDF/FTC or placebo	Follow-up 36 weeksLNG levels all above 400 pg/ml; mean LNG levels 469–660 pg/mlLNG levels lower for women randomized to the TDF/FTC arm, but in multivariable analysis TDF/FTC use not associated with changes in LNG levels compared with placebo	Strengths: TDF levels measured to assess for adherenceWeaknesses: Small sample size; percentage retention not stated; LNG concentration measures were missing for 42 time pointsFunded by government
Landolt *et al*. [15,19]Thailand	Open-label; nonrandomized clinical trialTo evaluate EE and ENG levels in women taking EFV-containing and NVP-containing cART and COCs	Forty-eight HIV+ nonpregnant, nonlactating women; 18–45 years, with regular menses, on EFV-containing or NVP-containing cART; no smoking, recent injectable contraceptive use, or contraindications to COCsForteen HIV− controls	COC-containing DSG for two cyclesNVP-containing or EFV-containing cART or no cART	NVP group: EE *C*_min_ ↓58% ENG *C*_min_↓22%EFV group: EE *C*_min_ ↓9% ENG *C*_min_↓61%One woman (6%) had NVP level below therapeutic level of 3.1 mg/lThree women had EFV level below therapeutic level of 1.0 mg/l	Strengths: prospective clinical trial; COCs and antiretrovirals at steady state; HIV+ womenWeaknesses: nonrandomized; small sample size; single measurement of ENG levels; high drop out/loss to follow-up rate; unable to measure ENG in 8/16 due to assay interferenceFunded by government
Vieira 2014 [34]Brazil	Prospective cohort with pharmacokinetic analysisTo evaluate the effects of EFV-containing or LPV/r-containing cART on ENG implant pharmacokinetics and to determine the impact of cART on luteal activity	Forty-five HIV+ women with regular menstrual cycles, BMI 18–30; excluded women with recent pregnancy or hormonal contraceptive use, acute infections or other opportunistic illnesses, drug or alcohol addiction, use of other potentially interacting drug, chronic diarrhea or malabsorption or noncompliance with cART	ENG implantLPV/r-containing or EFV-containing cART or no cART	ENG levels through 24 weeksEFV group: ENG AUC↓63% *C*_max_↓54% *C*_min_ ↓70%LPV/r group: ENG AUC↑52% *C*_max_ ↑61% *C*_min_ ↑ 34%	Strengths: Clearly described population and methods; valid assays, HIV+Weaknesses: Small sample size; nonrandomizedFunded by government
Atrio *et al*. [16]Atrio *et al*. [56]Dubois *et al*. [17]USA	Open-label; nonrandomized; clinical trialTo compare NET pharmacokinetic in women taking cART with PIs compared with women receiving other cART regimens	Thirty-five HIV+ women age 18–44 years; no changes in medications; no recent hormonal contraceptives; no immunocompromise; no liver or renal disease; normal ovulation; BMI <40; >30 days postpartum;	POPs containing NETIn PI-containing cART group: 11 taking ATV (10/11 on ATV/r); 3 DRV/r; 2 LPV/rIn control group: four women not taking PI-containing cART; 13 taking combinations including ETR, RPV, TDF, FTC, and RAL	PI group: NET AUC ↑50% NET *C*_max_ ↑33% NET *C*_min_ ↑26%In subanalysis limited to women on ATV/r;NETAUC ↑35% NET *C*_min_ ↑39% NET C24 ↑67%	Strengths: clearly described population and methods; valid assays; HIV+ womenWeaknesses: nonrandomized; small sample size; cART use self-reportedFunded by government
Crauwels *et al*. [27]UK	Open-label, three period pharmacokinetic studyTo evaluate the effect of RPV on COC pharmacokinetics and vice versa, and assess effects on sex hormones and safety of co-administration	Eighteen healthy nonsmoking women, 18–45 years; BMI 18–30 (median 24.6); 67% white; excluded pregnant, breast-feeding, or menopausal women, those with history of drug/alcohol abuse, skin disease, or any significant medical problems; use of concomitant medication	COC-containing NETIn third cycle; RPV 25 mg daily days 1–15	Thirteen completed trialEE *C*_min_ and AUC unchanged EE *C*_max_ ↑17%NET AUC; *C*_min_; *C*_max_ unchangedRPV pharmacokinetic unchanged from historical controls	Strengths: clearly described population and methods; valid assays; directly observed therapyWeaknesses: healthy women; short-term dosing; single antiretroviral; high discontinuation rateFunded by industry
Carten *et al*. [52]USA	Open-label two period pharmacokinetic studyTo determine the effect of EFV on the pharmacokinetics of LNG EC and vice versa, and assess safety	Twenty-four healthy women; 18–45; normal BMI (mean BMI 27) with no recent use of hormonal contraceptives or other interacting medications; women were either sterilized or used two nonhormonal contraceptive methods	LNG ECPs (0.75 mg) at 0 and 12 h on days 0 and 17EFV 600 mg 72 h after day 0, for 14 days.	Twenty-one women completed studyLNG AUC ↓58% LNG *C*_max_ ↓45% LNG *C*_min_ ↓69%	Strengths: clearly described population and methods; valid assaysWeaknesses: small sample size; healthy women; single antiretroviralFunded by industry
Piscitelli *et al*. [29]UK	Randomized crossover pharmacokinetic studyTo examine if GSK2248761 (fosdevirine) interacts with CYP450 substrates, including COCs	Ten healthy women, without hepatitis and not taking any medications	COC-containing DRSPGSK228761 200 mg or placebo days 1–11	EE levels unchangedDRSP AUC, *C*_max_, *C*_min_ ↑18–22%	Strengths: randomizedWeaknesses: short term administration; healthy women; single antiretroviral; small sample size; very few study details provided; trials of fosdevirine on hold due to other safety concerns; study terminated early for unknown reasonsFunded by industry
Kasserra *et al*. [57]USA	Randomized crossover pharmacokinetic studyTo determine the effect of vicriviroc alone or with RTV on COC pharmacokinetics and evaluate safety	Twenty-seven healthy nonpregnant women, 18–40 years; no recent medication use other than acetaminophen or oral contraceptives; BMI 19–32 (median 24.5)	COC-containing NETVicriviroc 75 mg BID for 10 days then vicriviroc 30 mg plus RTV 100 mg daily for next 11 days; group 2: RTV 100 mg daily for first 10 days then plus vicriviroc 30 mg daily for next 11 days	VCV alone: EE AUC and *C*_max_ unchanged NET AUC and *C*_max_ unchangedRTV alone: EE *C*_max_ ↓11% EE AUC ↓29% NET *C*_max_ ↓11% NET AUC ↓7%VCV+RTV: EE *C*_max_ ↓24%% EEAUC ↓29% NET: *C*_max_ ↓11% NET AUC ↓17%No severe or serious AEs	Strengths: randomized; valid assaysWeaknesses: healthy women; small sample size; single antiretroviralFunded by industry
Anderson *et al*. [61]USA	Randomized crossover pharmacokinetic studyTo assess the effect of RAL on COC pharmacokinetic	Twenty healthy nonobese nonpregnant women 18–45 years (mean age 27) using additional barrier contraceptive	COC-containing NGMRaltegravir 400 mg twice daily or placebo days 1–21	Nineteen women completed the trialEE levels unchangedNGMN AUC ↑ 14% NGMN *C*_max_ ↑29%.No serious clinical or laboratory AEs	Strengths: Randomized; placebo-controlled; clearly described population and methods; valid assaysWeaknesses: evening dose of RAL on day 21 of both periods missed; small sample size; healthy women; single antiretroviralFunded by industry
Zhang *et al*. [55]USA	Open-label three period pharmacokinetic studyTo assess the impact of RTV-boosted ATV on COC pharmacokinetic	Twenty healthy nonpregnant nonbreast-feeding women, 18–45 years (mean age 28); BMI 18–32 (mean 25)	COC containing NGMIn third cycle; ATV/r 300 mg/100 mg daily days 1–14	EE AUC ↓19% *C*_max_ ↓16% *C*_min_ ↓37%NGMN *C*_max_ ↑ 68% AUC ↑85% *C*_min_ ↑102%Dose normalization estimate magnitude of reduction with lower dose EEATV AUC ↑20% than historical controlsMore AEs with co-administration than with COCs alone (vomiting; headache and abdominal pain); no deaths or SAEs	Strengths: Clearly described population and methods; valid assaysWeaknesses: small sample size; healthy women; single antiretroviralFunded by industry
Stuart *et al*. [30]Malawi	Open label; nonrandomized clinical trialTo assess the feasibility of measuring anovulation in a pharmacokinetic study of COCs and antiretrovirals	Nine women ages 21–35 (3/group) with similar age and BMI: group 1 included HIV+ women on cART; group 2 included HIV+ women not on cART; and group 3 included HIV− women	COC with NGNVP-containing cART or no cART	LNG and EE levels measured by radioimmunoassayLNG AUC 147 in NVP group; 114 no cART group; and 38 in HIV− womenEE AUC; 1384, 1457, 1144, respectivelyAntiretroviral pharmacokinetic similar to historical controls	Strengths: Clearly described population and methods; valid assays; included HIV+ womenWeaknesses: small sample size; nonrandomized; progesterone measured only 1 dayFunding source not reported
Sevinsky *et al*. [26]USA	Open-label three period pharmacokinetic studyTo examine effect of EFV on pharmacokinetics of EE and NGMN and vice versa	Twenty-eight healthy women; 18–45 years (median 26); BMI 20–32 (median 25); on COCs for at least 2 months and no baseline safety issues or breakthrough bleeding	COC-containing NGMEFV 600 mg daily for 14 days during third cycle	Nineteen women completed studyEE AUC, *C*_max_, *C*_min_ unchangedNGMN *C*_max_ ↓46%; AUC ↓64% *C*_min_ ↓82%Posthoc LNG *C*_max_; AUC; and *C*_min_ ↓80–86%EFV levels comparable to historical controls	Strengths: clearly described population and methods; valid assaysWeaknesses: small sample size; healthy women; single antiretroviral; high discontinuation rateFunded by industry
Vogler *et al*. [37]USA	Open-label; nonrandomized; clinical trialTo evaluate pharmacokinetic interactions between LPV/r and contraceptive patch and COCs	Thirty-two nonpregnant; premenopausal HIV+ women >13 years either on stable LPV/r regimens for at least 14 days (*n* = 8) or not on cART or taking NRTIs only (*n* = 24); nonsmoking; median weight 72 kg; no recent use of injectables or COCs; <198 lb; not taking enzyme inducers	EE/NGMN contraceptive patchSingle-dose COC-containing NETLPV/r (400 mg/100 mg twice a day) or no cART/NRTIs only	Patch EE AUC ↓45% *C*_min_ ↓25%NGMN AUC ↑83% *C*_min_ ↑134%COC EE AUC ↓55%LPV AUC ↓19% *C*_min_ ↓27% *C*_max_ ↓22%RTV AUC↓24% *C*_min_ ↓14% *C*_max_ ↓8%	Strengths: clearly described population and methods; valid assays; HIV infectedWeaknesses: study closed prematurely due to slow accrual; small sample size; single dose COC; high loss to follow-up; not randomizedFunded by government
Schöller-Gyüre *et al*. [28]USA	Open-label three period pharmacokinetic studyTo assess the effect of ETR on COC pharmacokinetics	Twenty-four healthy women; 18–45 years (median 24); BMI 18–30; 97% white; nonsmoking; not pregnant or breast-feeding; no contraindications to hormonal contraceptives; not taking enzyme inducers	COC containing NETIn cycle 3; 200 mg ETR twice daily from day 1 to 15	EE AUC ↑ 22% *C*_max_ ↑33% *C*_min_ ↑9%NET *C*_max_; AUC unchanged *C*_min_ ↓22%ETR levels higher than historical controls	Strengths: Clearly described population and methods; valid assaysWeaknesses: small sample size; healthy women; single antiretroviral; not randomizedFunded by industry
Kearney and Mathias [58]USA	Open-label; two period pharmacokinetic studyTo evaluate the effect of TDF on the pharmacokinetic of COCs	Twenty healthy nonpregnant; nonlactating women; 19–45 years taking study COC for at least 3 months; without recent medication use or active alcohol or drug use; mean age 25 years; mean weight 64 kg	COC NGMTDF 300 mg/day on days 15–21 of contraceptive cycle 2	No change in NGMN and EE pharmacokineticTFV levels similar to historical dataNo serious AEs and no discontinuations due to AEs; AEs reported by 10 participants; most commonly headache; rash; dysmenorrhea; nausea; and rhinitis AEs reported by 10 participants; most commonly headache; rash; dysmenorrhea; nausea; and rhinitis	Strengths: clearly described population and methods; valid assaysWeaknesses: healthy women; single antiretroviral; small sample sizeFunded by industry
Abel *et al*. [60]USA	Randomized controlled; crossover pharmacokinetic studyTo evaluate the effect of MVC on COC pharmacokinetic and to assess pharmacokinetic and safety of MVC in women	Fifteen healthy sterilized white women; age 32–45; between 7 and 76 kg (mean 65 kg) and BMI 18–30	COC LNGMVC 100 mg twice daily or placebo days 1–10 and am of day 11	EE AUC; *C*_max_ unchangedLNG AUC; *C*_max_ unchangedMVC pharmacokinetic within the range seen in healthy males in previous studiesNo clinically significant abnormalities or severe or serious AEs	Strengths: randomized; placebo controlled; clearly described population and methods; valid assays; 100% retentionWeaknesses: small sample size; healthy women; single antiretroviral; COC only given days 2–8Funded by industry
Nanda *et al*. [32]Brazil	Open-label; nonrandomized; pharmacokinetic studyTo evaluate the effect of EFV-containing cART on pharmacokinetics of MPA and to determine effects on suppression of ovulation and bleeding patterns	Thirty-three HIV+ women aged 19–40 years; with regular menstrual cycles and BMI 18–30 kg/m^2^; not recently pregnant or breast-feeding	DMPA 150 mg given at enrollmentEFV-containing cART	No difference in MPA levels between groups through 12 weeks	Strengths: Clearly described population and methods; valid assays; HIV+ womenWeaknesses: small sample size; no EFV levels; no MPA levels beyond 12 weeksFunded by government
Sekar *et al*. [38]Belgium	Randomized crossover pharmacokinetic studyTo investigate the effect of DRV/r on COC pharmacokinetics and to examine safety	Twenty-two nonsmoking healthy women, 18–45 years; BMI 18–30; using a second nonhormonal contraceptive method	COC containing NETDRV/r 600/100 mg twice daily days 1–14 or no treatment	Eleven women completed studyEE AUC ↓44% *C*_min_ ↓62% *C*_max_ ↓32%NET AUC ↓14% *C*_min_ ↓30% *C*_max_ ↓10%DRV and RTV levels comparable to historical controls	Strengths: clearly described population and methods; valid assays; randomizedWeaknesses: small sample size; healthy women; single antiretroviral; high discontinuation rateFunded by industry
Cohn *et al*., Watts *et al*. [14,31]USA	Nonrandomized open-label pharmacokinetic studyTo evaluate the effect of various antiretrovirals on pharmacokinetics of MPA and vice versa; and to determine effects on suppression of ovulation and adverse events	Seventy-two HIV+ nonpregnant; premenopausal women, 22–46 years (median 35); median weight 71 kg; with no recent potentially interacting drugs; women required to use second nonhormonal method of contraception	DMPAAntiretrovirals regimens containing NFV; EFV; NVP; or no antiretroviral/NRTI only	No difference in MPA AUC; *C*_max_; *C*_min_; clearance half-life between groupsNVP AUC slightly higher after DMPA. No changes in EFV; nelfinavir pharmacokinetic	Strengths: clearly described population and methods; valid assays; HIV+ womenWeaknesses: nonrandomized; small sample sizeFunded by government
Aweeka *et al*. [65]USA	Open-label two-period pharmacokinetic time series studyTo investigate the effects of sex and contraceptives on ZDV pharmacokinetic and HIV viral load; to evaluate the effect of COCs and DMPA on plasma and genital HIV load among women on stable cART	HIV+: 18 men and 20 women; 22–52 years; on stable ZDV-containing cART	COC containing NET or DMPA begun at second cycle and continued through third cycleParticipants randomized to oral or intravenous ZDV (200 mg)Antiretrovirals included indinavir; nelfinavir; or any NRTI except d4T or tenofovir	Fourteen women (eight DMPA; six COC) provided pharmacokinetic dataNo effect on plasma or cervical HIV viral loadZDV levels and levels of by radioimmunoassay and liquid chromatography/mass spectrometryNo change in ZDV pharmacokinetic after contraceptive useNo differences between COC and DMPA on ZDV pharmacokinetic	Strengths: HIV+; prospective pharmacokinetic study; both PO and IV dosing of ZDVWeaknesses: open-label; nonrandomized; small sample; study stopped early due to slow enrollment; ZDV levels analyzed by two different methods due to discontinuation of reagents; no disaggregation of data for oral contraceptive vs. DMPAFunded by government
Burger *et al*. [62]Netherlands	Single time point retrospective pharmacokinetic analysisTo characterize factors that influence interpatient variability in EFV concentrations	Sixty-six HIV+ women	Eight hormonal contraceptive userscontaining EFV-containing cART	EFV concentration: No HC: 5.0 mg/l HC: 2.7 mg/l	Strengths: HIV+ womenWeaknesses: Study not designed to look at contraceptive effects; retrospective; single time point; very few hormonal users; population not well described; self-reported hormonal contraceptive use, type not specifiedFunding source not specified
Muro *et al*. [63]Netherlands	Open-label; single-period pharmacokinetic studyTo evaluate factors that influence interpatient variability in single dose NVP half-life	Forty-four healthy nonpregnant women age 18–40 (median 26 years); without hepatitis infection; median weight 64 kg	Seventeen oral contraceptive users 27 nonusersSingle dose of 200 mg of NVP	Seventeen women reported COCs; median time to first undetectable NVP plasma level was 21 days; longer than in 27 women not reporting COC use (14 days; *P* <0.001)Median half-life of NVP in COC users versus nonusers not significantly different (69.7 vs. 52.8 h; *P* = 0.053).	Strengths: clearly described population and methods; valid assaysWeaknesses: study not designed to look at contraceptive effects; few hormonal users; healthy women; single dose of single antiretroviral; self-reported hormonal contraceptive useFunding source not specified
Frohlich *et al*. [64]Germany	Open-label; two period pharmacokinetic studyTo investigate the influence of COCs on SQV pharmacokinetic and to assess the potential contribution of CYP3A4 and P-glycoprotein	Eight healthy nonsmoking nonpregnant women with regular menses; mean age 24 years and mean BMI 21; not using any potentially interacting drugs	COC containing GES days 4-25600 mg SQV on days 1 and 22	No effect of COCs on SQV pharmacokinetics	Strengths: Clearly described population and methods; valid assaysWeaknesses: not randomized; very small sample size; short course of COCs; healthy women; single antiretroviral only given twiceFunded by government
Mildvan *et al*. [53]USA	Open-label, single dose, two period pharmacokinetic studyTo determine the effects of NVP on COC pharmacokinetics and vice versa	Fourteen HIV+ nonpregnant, nonlactating, nonsmoking women; age18–65 (mean age 37); viral load <400; CD4^+^ cell count > 100 cells/μl; normal renal and hepatic function; no RTV or DLV use	Single dose of COC containing NET on cycle day 1 and 30NVP 200-mg daily on days 2–15; then 200-mg twice daily days 16–29; single dose on day 30cART regimens included IDV; NFV; SQV/RTV	Ten women completed the studyEE AUC ↓29% *C*_max_ unchangedNET AUC ↓18% *C*_max_ unchangedNVP levels similar to historical controls	Strengths: HIV+ clearly described population and methods; valid assaysWeaknesses: small study; only single dose COC; NVP added to current cART regiment; included postmenopausal womenFunded by industry
Ouellet *et al*. [54]Canada	Single dose, single period pharmacokinetic studyTo assess the effects of RTV on EE pharmacokinetics	Twenty-three healthy nonpregnant nonlactating women, 18–45, close to ideal weight; women were postmenopausal, sterilized, practiced abstinence, or had a vasectomized partner	Single dose of COC with 50 μg EE + 1 mg ethynodiol diacetate given on cycle days 1 and 29RTV oral solution from day 15–30, 300 mg q12h on Day 15, 400 mg q12h on Day 16, and 500 mg q12h thereafter	EE *C*_max_ ↓32% AUC ↓41%	Strengths: valid assaysWeaknesses: no progestin levels; nonrandomized; single dose COC; postmenopausal healthy women; nonstandard RTV dosesFunded by industry

Abbreviations for antiretrovirals and contraceptive steroids defined in Tables 1 and 2. AUC, area under the curve; *C*_max_, Peak concentration; *C*_min_, rough concentration; COC, combined oral contraceptive; DMPA, depot medroxyprogesterone acetate; ECP, emergency contraceptive pill; MPA, medroxyprogesterone acetate; POP, progestin-only pill.

**Table 5:** Summary of included clinical and pharmacokinetic data on HC-antiretroviral drug interactions; PK differences <30% considered no change (↔), blank cells indicate absence of data.

Antiretroviral	Ethinyl estradiol pharmacokinetics	Progestin pharmacokinetics	Antiretroviral pharmacokinetics	Contraceptive effectiveness (pregnancy rates per 100 woman-years or ovulation %)	Antiretroviral effectiveness	Reported adverse events/toxicity
NNRTIs						
Efavirenz						
COCs	AUC↔	ENG AUC ENG C24↓61% NGM AUC↓64%	EFV levels <1.0 mg/l in 3/16	Pregnancy rates 13–15		Bleeding, nausea, mood change, dizziness
	C24↔	NGM *C*_max_↓46%		Ovulation rates 0–19		
		NGM *C*_min_↓82%				
ECPs		LNG AUC ↓58%				
		LNG *C*_max_ ↓45%				
		LNG *C*_min_ ↓69%				
Injectables	NA	MPA ↔	↔	Pregnancy rates 4–9	No effect on time to virologic suppression, CD4^+^ cell count, death, viral load, treatment failure	No grade3/4 AEs
				Ovulation rate 0		
LNG implants		LNG AUC ↓47%		Pregnancy rates 6–15	No change in CD4^+^ cell count or HIV viral load	Bleeding
ENG implants		ENG AUC↓63%		Pregnancy rate 5		
		ENG *C*_max_↓54%		Ovulation rate 3		
		ENG *C*_min_ ↓70% in				
Nevirapine						
COCs	EE AUC↔	NET AUC↔	↔	Pregnancy rates 6–10		Nausea, headache, breast tenderness, mood changes
	EE *C*_max_ ↔	NET *C*_max_↔		Ovulation rates 0–30		
	EE C24 ↓58%	LNG AUC↔				
		LNG *C*_min_↔				
		ENG C24↔				
Injectables			↔	Pregnancy rates 3–8		No grade 3 or 4 AEs
LNG implants				Pregnancy rate 0–2		
ENG implants				Pregnancy rate 2		
Etravirine						
COCs	AUC↔	NET *C*_max_↔	↔	Ovulation rate 0		Nine discontinued due to AEs (rash in 7)
	EE *C*_max_ ↑ 33%,	NET AUC↔				
	*C*_min_↔	NET *C*_min_↔				
Rilpivirine						
COCs	*C*_max_ ↔	NET *C*_max_↔	↔	Ovulation rate 0		No grade 3 or 4 AEs
	*C*_min_ ↔	NET *C*_min_↔				
	AUC ↔	NET AUC↔				
Ritonavir-boosted PIs						
Lopinavir/ ritonavir						
COCs, patch	EE AUC ↓55%	Patch:	↔	COC pregnancy rate 15		One grade 3 AE
	Patch:	NGMN AUC ↑ 83%, NGMN *C*_min_ ↑ 134%		Patch ovulation rate 0		
	EE AUC ↓ 45%					
	EE *C*_min_ ↔					
Injectables		MPA AUC ↑46%	↔	Pregnancy rate 5–7	No difference in CD4^+^ cell count or HIV RNA	Menstrual irregularities in 25%, only one considered grade 3 due to persistent bleeding
		MPA *C*_max_ ↑66%		Ovulation rate 0		
LNG implants				Pregnancy rate 0		
ENG implants		AUC ↑ 52%		Pregnancy rate 1		
		*C*_max_ ↑ 61%		Ovulation rate 0		
		*C*_min_ ↑ 34%				
Atazanavir/ ritonavir						
COCs	AUC ↔	NGM *C*_max_ ↑ 68%, NGM AUC ↑ 85%, NGM *C*_min_ ↑ 102%,	↔			incr side-effects
	*C*_max_ ↔					
	*C*_min_ ↓37%					
POPs		NET *C*_max_ ↑ 39%		Cervical mucus remained thickened		
		NET C24 ↑ 67%,				
		NET AUC ↑ 50%				
Darunavir/ ritonavir						
COCs	*C*_min_ ↓62%	NET *C*_min_ ↓30%	DRV and RTV ↔			26% discontinued due to grade two AEs
	*C*_max_ ↓32%	NET *C*_max_ ↔				
	AUC ↓44%;	NET AUC ↔				
SQV/ritonavir						
COCs			↔			
PIs without ritonavir						
Indinavir						
COCs				Zero pregnancies		
Nelfinavir						
COCs				Higher failure rate with NFV than other PIs		
Injectables		MPA ↔	AUC ↔	Zero pregnancies	No change in CD4^+^ cell count or HIV RNA	No grade 3 or 4 AEs
			*C*_max_ ↔	Ovulation rate 0		
			*C*_min_ ↓46%			
CCR5 antagonists						
Maraviroc						
COCs	↔	LNG ↔				No serious AEs
Integrase inhibitors						
Dolutegravir						
COCs	↔	↔				
Raltegravir						
COCs	↔	↔				
NRTIs						
Tenofovir disaproxil fumarate, emtricitabine						
COCs	↔	↔		Pregnancy rate 18–35		
Injectables				Pregnancy rate 2–5	No difference	
LNG implants				Pregnancy rate <1		
Zidovudine						
COCs		↔			No change in plasma and cervical HIV viral load	
Injectables		↔			No change in plasma and cervical HIV viral load	

Abbreviations for antiretrovirals and contraceptive steroids defined in Tables 1 and 2. COC, combined oral contraceptive; DMPA, depot medroxyprogesterone acetate; ECP, emergency contraceptive pill; POP, progestin-only pill. AUC, area under the curve; *C*_max_, peak concentration; *C*_min_, trough concentration.
