# Supplementary material for: Drug interactions between hormonal contraceptives and antiretrovirals
Source: AIDS. 2017 Apr 3;31(7):917–52. doi: 10.1097/QAD.0000000000001392 (PMC5378006; doi:10.1097/QAD.0000000000001392)
Supplement: Supplemental Digital Content [file aids-31-0917-s001.doc]

**Search Strategy Supplement**

PubMed, Embase, CENTRAL, and POPLINE databases were searched from inception through the listed dates for potentially relevant publications.

PubMed was searched on September 21 2015 using ((hormonal AND contracepti*) OR ("hormonal methods")) OR ((progestin* OR progestins OR Progesterone OR progestogen* OR progestagen*) AND contracept*) OR (oral contracept*) OR ((((depo OR depot) AND medroxyprogesterone) OR depo medroxyprogesterone OR depo OR depot OR dmpa OR "net en" OR net-en OR "norethisterone enanthate" OR norethisterone-enanthate OR Medroxyprogesterone 17-Acetate) AND (contracept* OR inject*)) OR (((levonorgestrel OR etonogestrel) AND implant) OR (uniplant OR jadelle OR implanon OR norplant OR norplant2 OR sino-implant)) OR (contraceptives, postcoital OR (contracept* AND (emergency OR postcoital OR "post coital")) OR "ulipristal acetate" OR "Plan B" OR mifepristone) OR ((levonorgestrel AND (intrauterine devices OR iud OR iucd OR ius OR "intrauterine system" OR "intra-uterine system" OR "intrauterine device" OR "intra-uterine device")) OR mirena) OR ((combin* AND inject* AND contracept*) OR (("once a month" OR monthly) AND inject* AND contracept*) OR (cyclofem OR lunelle OR mesigyna OR "cyclo provera" OR cycloprovera)) OR ((((contraceptive devices OR contraceptive agents) AND ring) OR nuvaring OR "nuva ring")) OR ((((contraceptive devices OR contraceptive agents) AND patch) OR "ortho evra" OR ortho evra) OR (desogestrel OR drospirenone OR ethinyl estradiol OR etonogestrel OR levonorgestrel OR norethindrone OR norgestrel OR norgestimate)) AND (("HIV Seropositivity" OR "HIV" OR "HIV Infections" OR "Acquired Immunodeficiency Syndrome") OR antiretroviral OR (abacavir OR amprenavir OR atazanavir OR cobicistat OR delavirdine OR didanosine OR darunavir OR dolutegravir OR efavirenz OR elvitegravir OR emtricitabine OR enfuvirtide OR etravirine OR fosamprenavir OR indinavir OR lamivudine OR lopinavir OR maraviroc OR nelfinavir OR nevirapine OR raltegravir OR rilpivirine OR ritonavir OR saquinavir OR stavudine OR tipranavir OR tenofovir OR zidovudine)).

Embase was searched on September 21 2015 using a longer strategy and a shorter strategy. The longer strategy used all of the following terms; ((hormonal AND contracepti*) OR ("hormonal methods")) OR oral contraceptive agent OR injectable contraceptive agent OR  ((progestin* OR progestins OR Progesterone OR progestogen* OR progestagen*) AND contracept*) OR (oral contracept*) OR ((((depo OR depot) AND medroxyprogesterone) OR depo medroxyprogesterone OR depo OR depot OR dmpa OR "net en" OR net-en OR "norethisterone enanthate" OR norethisterone-enanthate OR Medroxyprogesterone 17-Acetate) AND (contracept* OR inject*)) OR (((levonorgestrel OR etonogestrel) AND implant) OR (uniplantOR jadelle OR implanon OR norplant OR norplant2 OR sino-implant)) OR (contraceptives, postcoital OR (contracept* AND (emergency OR postcoital OR "post coital")) OR "ulipristal acetate" OR "Plan B" OR mifepristone) OR ((levonorgestrel AND (intrauterine devices OR iud OR iucd OR ius OR "intrauterine system" OR "intra-uterine system" OR "intrauterine device" OR "intra-uterine device")) OR mirena) OR ((combin* AND inject* AND contracept*) OR (("once a month" OR monthly) AND inject* AND contracept*) OR (cyclofem OR lunelle OR mesigyna OR "cyclo provera" OR cycloprovera)) OR ((((contraceptive devices OR contraceptive agents) AND ring) OR nuvaring OR "nuva ring")) OR ((((contraceptive devices OR contraceptive agents) AND patch) OR "ortho evra" OR ortho evra) OR (desogestrel OR drospirenone OR ethinyl estradiol OR etonogestrel OR levonorgestrel OR norethindrone OR norgestrel OR norgestimate)) AND (("HIV Seropositivity" OR "HIV" OR "HIV Infections" OR "Acquired Immunodeficiency Syndrome" OR human immunodeficiency virus) OR (abacavir OR amprenavir OR atazanavir OR delavirdine OR didanosine OR darunavir OR efavirenz OR elvitegravir OR emtricitabine OR etravirine OR fosamprenavir OR indinavir OR lamivudine OR lopinavir OR maraviroc OR nelfinavir OR nevirapine OR raltegravir OR rilpivirine OR ritonavir OR saquinavir OR stavudine OR tipranavir OR tenofovir OR zidovudine)). The shorter search strategy used for the Embase database included contracept* AND ('art'/exp/mj OR art OR antiretroviral* OR 'hiv'/exp/mj OR hiv) AND ('drug interaction'/exp/mj OR 'drug interaction') AND [embase]/lim. Articles from both searches were reviewed for relevance.

CENTRAL database was searched on September 21 2015 and included the following many terms (((hormonal AND contracepti*) OR ("hormonal methods")) OR ((progestin* OR progestins OR Progesterone OR progestogen* OR progestagen*) AND contracept*) OR (oral contracept*) OR ((((depo OR depot) AND medroxyprogesterone) OR depo medroxyprogesterone OR depo OR depot OR dmpa OR "net en" OR neten OR "norethisterone enanthate" OR norethisteroneenanthate OR Medroxyprogesterone 17Acetate) AND (contracept* OR inject*)) OR (((levonorgestrel OR etonogestrel) AND implant) OR (uniplantOR jadelle OR implanon OR norplant OR norplant2 OR sinoimplant)) OR (contraceptives, postcoital OR (contracept* AND (emergency OR postcoital OR "post coital")) OR "ulipristal acetate" OR "Plan B" OR mifepristone) OR ((levonorgestrel AND (intrauterine devices OR iud OR iucd OR ius OR "intrauterine system" OR "intrauterine system" OR "intrauterine device" OR "intrauterine device")) OR mirena) OR ((combin* AND inject* AND contracept*) OR (("once a month" OR monthly) AND inject* AND contracept*) OR (cyclofem OR lunelle OR mesigyna OR "cyclo provera" OR cycloprovera)) OR ((((contraceptive devices OR contraceptive agents) AND ring) OR nuvaring OR "nuva ring")) OR ((((contraceptive devicesMeSH OR contraceptive agents) AND patch) OR "ortho evra" OR ortho evra) OR (desogestrel OR drospirenone OR ethinyl estradiol OR etonogestrel OR levonorgestrel OR norethindrone OR norgestrel OR norgestimate))) AND (("HIV Seropositivity" OR "HIV" OR "HIV Infections" OR "Acquired Immunodeficiency Syndrome") OR (abacavir OR amprenavir OR atazanavir OR delavirdine OR didanosine OR darunavir OR efavirenz OR elvitegravir OR emtricitabine OR etravirine OR fosamprenavir OR indinavir OR lamivudine OR lopinavir OR maraviroc OR nelfinavir OR nevirapine OR raltegravir OR rilpivirine OR ritonavir OR saquinavir OR stavudine OR tipranavir OR tenofovir OR zidovudine)) AND (drug interaction OR drug interactions OR drug interacting OR drugs interaction OR drugs interactions OR drugs interacting) in Title, Abstract, Keywords. A shorter stretgey for CENTRAL was also used that included only contracept* AND (ART OR antiretrovial therapy OR antiretroviral treatment) in Title, Abstract, Keywords.

POPLINE was searched on 5 Sept 2015 using terms ((hormonal AND contracepti*) OR ("hormonal methods")) OR ((progestin* OR progestins OR Progesterone OR progestogen* OR progestagen*) AND contracept*) OR (oral contracept*) OR ((((depo OR depot) AND medroxyprogesterone) OR depo medroxyprogesterone OR depo OR depot OR dmpa OR "net en" OR neten OR "norethisterone enanthate" OR norethisteroneenanthate OR Medroxyprogesterone 17Acetate) AND (contracept* OR inject*)) OR (((levonorgestrel OR etonogestrel) AND implant) OR (uniplantOR jadelle OR implanon OR norplant OR norplant2 OR sinoimplant)) OR (contraceptives, postcoital OR (contracept* AND (emergency OR postcoital OR "post coital")) OR "ulipristal acetate" OR "Plan B" OR mifepristone) OR ((levonorgestrel AND (intrauterine devices OR iud OR iucd OR ius OR "intrauterine system" OR "intrauterine system" OR "intrauterine device" OR "intrauterine device")) OR mirena) OR ((combin* AND inject* AND contracept*) OR (("once a month" OR monthly) AND inject* AND contracept*) OR (cyclofem OR lunelle OR mesigyna OR "cyclo provera" OR cycloprovera)) OR ((((contraceptive devices OR contraceptive agents) AND ring) OR nuvaring OR "nuva ring")) OR ((((contraceptive devicesMeSH OR contraceptive agents) AND patch) OR "ortho evra" OR ortho evra) OR (desogestrel OR drospirenone OR ethinyl estradiol OR etonogestrel OR levonorgestrel OR norethindrone OR norgestrel OR norgestimate)) AND (("HIV Seropositivity" OR "HIV" OR "HIV Infections" OR "Acquired Immunodeficiency Syndrome") OR (abacavir OR amprenavir OR atazanavir OR delavirdine OR didanosine OR darunavir OR efavirenz OR elvitegravir OR emtricitabine OR etravirine OR fosamprenavir OR indinavir OR lamivudine OR lopinavir OR maraviroc OR nelfinavir OR nevirapine OR raltegravir OR rilpivirine OR ritonavir OR saquinavir OR stavudine OR tipranavir OR tenofovir OR zidovudine)) AND drug interaction OR drug interactions OR drug interacting OR drugs interaction OR drugs interactions OR drugs interacting.
